# Dasatinib and CAR T-Cell Therapy in Newly Diagnosed Philadelphia Chromosome–Positive Acute Lymphoblastic Leukemia: A Nonrandomized Clinical Trial

**DOI:** 10.1001/jamaoncol.2025.0674

**Published:** 2025-04-17

**Authors:** Mingming Zhang, Shan Fu, Jingjing Feng, Ruimin Hong, Guoqing Wei, Houli Zhao, Mengyu Zhao, Huijun Xu, Jiazhen Cui, Simao Huang, Xiaoyu Wu, Lianxuan Liu, Jie Sun, Wenjun Wu, Yuanyuan Zhu, Jingsong He, Yi Zhao, Zhen Cai, Weiyan Zheng, Xiujin Ye, Jimin Shi, Yi Luo, Dongrui Wang, Alex H. Chang, Yongxian Hu, He Huang

**Affiliations:** 1 Bone Marrow Transplantation Center, the First Affiliated Hospital, and Liangzhu Laboratory, Zhejiang University School of Medicine, Hangzhou, China; 2 Institute of Hematology, Zhejiang University, Hangzhou, China; 3 Zhejiang Province Engineering Research Center for Stem Cell and Immunity Therapy, Hangzhou, China; 4 Engineering Research Center of Gene Technology, Ministry of Education, Institute of Genetics, School of Life Sciences, Fudan University, Shanghai, China; 5 Shanghai YaKe Biotechnology, Shanghai, China

## Abstract

**Importance:**

A combination of tyrosine kinase inhibitors and chimeric antigen receptor (CAR) T cells has made a breakthrough in refractory or relapsed Philadelphia chromosome (Ph)–positive acute lymphoblastic leukemia (ALL). However, it remains unclear if this treatment in newly diagnosed Ph-positive ALL is associated with high rates of complete molecular remission (CMR) and leukemia-free survival.

**Objective:**

To evaluate the efficacy and safety of dasatinib in combination with CAR T cells as frontline therapy in adults with newly diagnosed Ph-positive ALL.

**Design, Setting, and Participants:**

This trial was conducted at a single center, the First Affiliated Hospital of Zhejiang University School of Medicine. Patients were enrolled in this phase 2, single-arm nonrandomized clinical trial between March 5, 2021, and April 13, 2024. The data cutoff date was February 10, 2025. The data analysis was conducted on February 11, 2025. The median duration of follow-up was 23.9 (range, 7.3-47.7) months. A total of 29 adults with newly diagnosed Ph-positive ALL and adequate organ function were screened for eligibility, and 1 patient who received a diagnosis of blast-phase chronic myeloid leukemia was excluded.

**Intervention:**

Dasatinib was administered with a 2-week vindesine and dexamethasone regimen as induction, followed by sequential CD19 and CD22 CAR T-cell therapies and single-agent dasatinib maintenance.

**Main Outcomes and Measures:**

The primary end point was CMR rate after CD19 CAR T-cell therapy. CMR was defined as undetectable *BCR/ABL1* transcripts as measured by quantitative reverse transcription polymerase chain reaction with a sensitivity of 10^−4^ in the bone marrow.

**Results:**

Twenty-eight patients (median [range] age, 48.5 [18.0-69.0] years; 10 female individuals [36%]) were enrolled, and 1 patient withdrew after induction. The CMR rate was 25% (7 of 28) after induction and increased to 85% (23 of 27) after CD19 CAR T-cell therapy. Twenty-five patients (89.3%) received sequential CD22 CAR T-cell therapy, and the CMR rate was 76% (19 of 25). Of the 52 CAR T-cell therapies, only 21 cases of grade 1 cytokine release syndrome occurred. After a median follow-up of 23.9 (range, 7.3-47.7) months, the 2-year overall survival and leukemia-free survival were 92%.

**Conclusions and Relevance:**

The results of this nonrandomized clinical trial suggest that the combination of dasatinib and CAR T-cell therapy showed encouraging efficacy in newly diagnosed Ph-positive ALL with acceptable toxic effects. Further studies with larger cohorts and longer follow-up durations are needed.

**Trial Registration:**

ClinicalTrials.gov Identifier: NCT04788472

## Key Points

**Question:**

What are the efficacy and safety of chimeric antigen receptor (CAR) T-cell therapy in combination with dasatinib as frontline therapy in adults with newly diagnosed Philadelphia chromosome (Ph)–positive acute lymphoblastic leukemia (ALL)?

**Findings:**

In this phase 2, single-arm, nonrandomized clinical trial that included 28 adults with Ph-positive ALL, the complete molecular remission rate after CD19 CAR T-cell therapy was 85%. This treatment showed good safety profile, with only grade 1 cytokine release syndrome and no neurotoxic effects.

**Meaning:**

The results of this trial suggest that the combination of CAR T-cell therapy and dasatinib was associated with encouraging efficacy and acceptable toxic effects as frontline therapy in adults with newly diagnosed Ph-positive ALL.

## Teaser

This nonrandomized clinical trial examines the efficacy and safety of dasatinib in combination with chimeric antigen receptor T cells as frontline therapy in adults with newly diagnosed Philadelphia chromosome–positive acute lymphoblastic leukemia.

## Introduction

The addition of tyrosine kinase inhibitors (TKIs) to chemotherapy has substantially improved outcomes in adults with Philadelphia chromosome (Ph)–positive acute lymphoblastic leukemia (ALL).^1,2^ Despite improved complete remission rates, the long-term survival remains modest, with a 5-year overall survival (OS) rate of less than 50%, which is attributed to the low rate of complete molecular remission (CMR).^3^

Immunotherapies, including bispecific antibodies,^4^ antibody-drug conjugates,^5^ and chimeric antigen receptor (CAR) T cells,^6^ have improved outcomes of refractory or relapsed (r/r) B-cell ALL. This has led to studies combining immunotherapies, such as blinatumomab, with TKIs in the frontline treatment of Ph-positive ALL.^7,8,9,10^ This chemotherapy-free strategy has been associated with high rates of molecular response and survival. CAR T-cell therapy achieved a CMR rate of 67.9% in r/r Ph-positive ALL,^11^ but to our knowledge has not yet been used for patients with a new diagnosis. This study aimed to evaluate the efficacy and safety of dasatinib in combination with CAR T cells as frontline therapy in adults with newly diagnosed Ph-positive ALL.

## Methods

This single-arm, phase 2 trial was conducted at the First Affiliated Hospital of Zhejiang University School of Medicine (the trial protocol is available in Supplement 1). This study was approved by the ethics committee of the hospital. Written informed consent was obtained from all patients. The data cutoff date was February 10, 2025. The data analysis was conducted on February 11, 2025.

The trial design is detailed in eFigure 1 in Supplement 2, with the treatment process and CAR T-cell manufacture outlined in the eMethods in Supplement 2. Briefly, patients underwent a 2-week induction with vindesine, dexamethasone, and continuous dasatinib (100 mg/d). Those achieving complete hematological remission (CHR) received sequential CD19 and CD22 CAR T-cell therapies, which were associated with reduced relapse in r/r B-cell ALL.^12^ The timing of CD22 CAR T included normal B-cell reappearance or failure to achieve CMR post CD19 CAR T-cell therapy. After lymphodepletion with fludarabine and cyclophosphamide, CAR T cells were administered at a target dose of 2 × 10^6^ cells/kg. All patients received dasatinib maintenance (100 mg per day). Intrathecal chemotherapy was administered twice on the first day of each lymphodepletion.

The primary end point was CMR after CD19 CAR T-cell therapy. Secondary end points were CMR after CD22 CAR T-cell therapy, leukemia-free survival (LFS), OS, the safety profile, and leukemia relapse characteristics. The definitions of CHR, CMR, major molecular remission, and molecular relapse are described in the eMethods in Supplement 2.

The sample size was calculated via PASS, version 15.0 (NCSS), using a single-stage phase 2 trial design. Targeting an improvement in CMR rate from 35%^7^ (postinduction) to 65%^11^ (post–CD19 CAR T-cell therapy), 24 evaluable patients were required (α = .05, 1-sided, 90% power) to reject a null hypothesis (CMR rate post–CD19 CAR T cell ≤35%) if the true rate was 65% or greater. The critical value was 13 or more CMR responders among 24 patients. Accounting for a 10% dropout rate, 27 patients were needed. CMR rates with 95% CIs were calculated using the Clopper-Pearson method. Continuous variables were compared using the Wilcoxon signed-rank test. Distributions of time-to-event data were estimated using the Kaplan-Meier method, and hazard ratios with 95% CIs were calculated using the Cox proportional hazards model. A log-rank test was used to compare differences between subgroups. Patients without events were censored at the final follow-up. Data were analyzed using R software (version 3.6.2; R Foundation), with statistical significance set at a 2-tailed *P* < .05.

## Results

### Patient Characteristics

Twenty-eight patients were enrolled between March 5, 2021, and April 13, 2024 (Figure; median [range] age, 48.5 [18–69] years; 18 male individuals [64%]; 16 [57%] p190, and 11 [39%] p210; Table 1). The data cutoff date was February 10, 2025.

**Figure.**
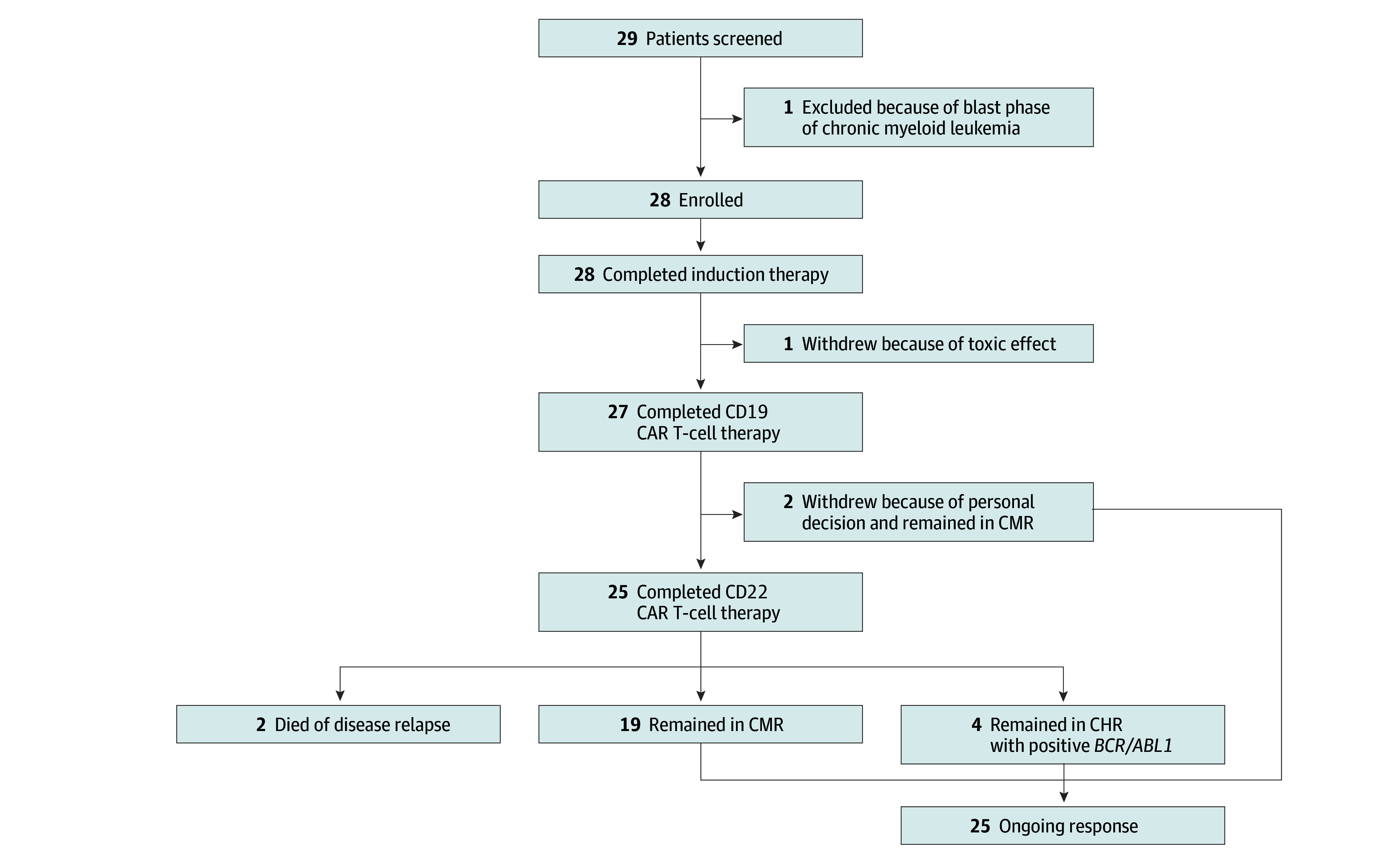
CONSORT Diagram CAR indicates chimeric antigen receptor; CHR, complete hematological remission; CMR, complete molecular remission.

**Table 1.** Baseline Characteristics

Characteristic	No. (%) (n = 28)
Age, median (range), y	48.5 (18.0-69.0)
Sex	
Female	10 (36)
Male	18 (64)
White blood cell counts at diagnosis, median (range), /μL	19 400 (1700-553 700)
Fusion protein	
p190	16 (57)
p210	11 (39)
Rare type	1 (4)
Additional genetic abnormalities	
*IKZF1* alterations	6 (21)
*PAX5* rearranged	2 (7)
*ZNF384* rearranged	1 (4)
Hypodiploidy	1 (4)
*CDKN2A/B* deletion	1 (4)
*KMT2D* variant	2 (7)
*RUNX1* alteration	2 (7)
Time between pretreatment to initiation of dasatinib, median (range), d	4.5 (1-8)
Time between initiation of induction to apheresis, median (range), d	41 (29-56)
Time between apheresis to CD19 CAR T-cell infusion, median (range), d	15 (8-97)^a^
Time between 2 infusions, median (range), d (n = 25)	88 (59-186)

Abbreviation: CAR, chimeric antigen receptor.

SI conversion factor: to convert white blood cell counts to ×10^9^/L, multiply by 0.001.

^a^ One patient was delayed in receiving CD19 CAR T cells because of a grade 3 adverse event of pulmonary infection during induction. The remaining 26 patients all received CD19 CAR T-cell infusion within 30 days after apheresis.

### Response

All 28 patients completed induction, achieving a 100% CHR rate (95% CI, 88%-100%) and a 25% CMR rate (7 of 28; 95% CI, 11%-45%; Table 2). One patient in CHR withdrew due to dasatinib toxic effects and was lost to follow-up.

**Table 2.** Clinical Response

Assessment	No./total No. (%)		
	CHR	CMR	MMR
After induction	28/28 (100)	7/28 (25)	3/28 (11)
After CD19 CAR T-cell therapy	27/27 (100)	23/27 (85)	2/27 (7)
After CD22 CAR T-cell therapy	25/25 (100)	19/25 (76)	1/25 (4)
At data cutoff date	25/27 (93)	21/27 (78)	3/27 (11)

Abbreviations: CAR, chimeric antigen receptor; CHR, complete hematological remission; CMR, complete molecular remission; MMR, major molecular remission.

Twenty-seven patients received CD19 CAR T-cell therapy and were included in the efficacy analysis. After CD19 CAR T-cell therapy, 23 of 27 (85%; 95% CI, 66%-96%) achieved CMR (Table 2), significantly exceeding the prespecified critical value (*P* < .001), leading to a rejection of the null hypothesis. Two patients withdrew due to personal decisions but remained in sustained CMR with dasatinib maintenance. Three molecular relapses occurred post–CD19 CAR T-cell therapy, 1 with *T315I* variant in *ABL1.*

Twenty-five patients completed sequential CD22 CAR T-cell therapy, of whom 18 were due to normal B-cell reappearance, and 7 were due to positive *BCR/ABL1*. The CMR rate post–CD22 CAR T-cell therapy was 76% (19 of 25; 95% CI, 55%-91%; Table 2).

### Relapse and Long-Term Survival

The median follow-up was 23.9 (range, 7.3–47.7) months. Two hematological relapses occurred. Both patients had *IKZF1* deletions at baseline, 1 with CD19^dim^ CD22-positive relapse without an *ABL1* variant, and the other with CD19-positive CD22-positive relapse accompanied by variants in *ABL1* (*F317L* and *Y253H*). Both patients died of disease progression. At the data cutoff date, 21 (78%; 95% CI, 58%-91%) patients remained in CMR, and 4 patients remained in durable *BCR/ABL1*–positive CHR (range, 11.9-42.8 months). The 2-year OS and LFS were 92% (95% CI, 82%-100%; eFigure 2 in Supplement 2). Inferior OS (100% vs 66.7%; *P* = .01) and LFS (100% vs 66.7%; *P* = .01) were observed among patients with *IKZF1* alterations (eFigure 3 in Supplement 2). Only the patient with a *T315I* variant in *ABL1* received consolidative allogeneic stem cell transplant (allo-HSCT).

### Safety

The treatment exhibited a good safety profile (eTable 1 in Supplement 2). The most common grade 3 or higher adverse events were hematological that were thought to be associated with the induction and lymphodepletion. Of the 52 CAR T-cell therapies, only 21 cases of grade 1 cytokine release syndrome occurred, and no immune effector cell–associated neurotoxicity syndrome was observed.

### CAR T-Cell Expansion

CAR T cells expanded well (eFigure 4 in Supplement 2). The peak numbers of CD19 CAR T cells (median [range], 492 [5-2001]/μL) were significantly higher than those of CD22 CAR T cells (50 [range, 3-1053]/μL; *P* = .03). The median time to reach peak expansion of CD19 and CD22 CAR T cells were 11 (range, 6-17) and 10 (range, 6-21) days, respectively.

## Discussion

To our knowledge, this was the first trial to evaluate the efficacy and safety of CAR T cells and dasatinib as frontline therapy for newly diagnosed Ph-positive ALL. The results showed this treatment was associated with high CMR and LFS rates with a good safety profile.

In recent years, several trials^7,8,9,10^ have investigated TKIs with blinatumomab as frontline therapy for Ph-positive ALL with molecular response rates of 60% to 87% and 3-year LFS rates higher than 75%. Moreover, the rates of allo-HSCT were very low. Our study showed that CAR T cells as a frontline therapy for Ph-positive ALL had comparable outcomes with blinatumomab, with a CMR rate of 85% and 2-year LFS rate of 92%. Only 1 patient received consolidative allo-HSCT.

Central nervous system (CNS) relapse was an issue of concern in first-line therapy with blinatumomab and dasatinib even with 8 or 12 doses of intrathecal chemotherapy.^7,8,10^ CAR T cells were able to cross the blood-brain barrier when used to treat CNS leukemia.^13,14^ Therefore, we hypothesized that CAR T cells may have a preventive association with CNS relapse. In our study, despite only 2 doses of intrathecal chemotherapy, no CNS relapse was observed. Longer follow-up is needed to observe the preventive association of CAR T cells with CNS leukemia.

### Limitations

The main limitations included a short follow-up and relatively small sample size; continued monitoring and larger future studies are warranted.

## Conclusions

In this nonrandomized clinical trial, the combination of dasatinib and CAR T-cell therapy showed encouraging efficacy and good safety in newly diagnosed Ph-positive ALL.

## Notes

Supplement 1. Trial protocol

Supplement 2. **eFigure 1.** Trial design **eFigure 2.** Overall survival and leukemia-free survival **eFigure 3.** Overall survival and leukemia-free survival, according to the presence or absence of *IKZF1* alterations **eFigure 4.** In vivo kinetics of CAR T cells **eTable.** Adverse events

Supplement 3. Data sharing statement
