# Supplementary material for: Dasatinib and CAR T-Cell Therapy in Newly Diagnosed Philadelphia Chromosome–Positive Acute Lymphoblastic Leukemia: A Nonrandomized Clinical Trial
Source: JAMA Oncol. 2025 Apr 17;11(6):625–9. doi: 10.1001/jamaoncol.2025.0674 (PMC12006910; doi:10.1001/jamaoncol.2025.0674)
Supplement: Supplement 1. — Trial protocol [file jamaoncol-e250674-s001.pdf]

Clinical Study of CAR-T Cell Therapy for Newly Diagnosed Ph-Positive B-Cell Acute Lymphoblastic Leukemia in Adults

Clinical Study Protocol

Protocol version number: V2.0

Protocol version date: December 24, 2020

Clinical study unit: The First Affiliated Hospital of Zhejiang University School of Medicine

Principal investigator: Prof. Huang He

December 24, 2020

Protocol revision history

releases	dates	Imprint
V1.0	2020-11-22	first draft
V2.0	2020-12-24	revised draft

Researcher's statement and signature page

The study is a prospective clinical study and the methodology, equipment and drugs used in the study have been widely used in clinical practice. A strict adverse event monitoring system will be set up in this study, and all adverse events will be carefully recorded and dealt with in a timely and effective manner. In the event of any serious or significant adverse events, whether or not they are related to the study intervention and whether or not the intervention has been performed, the study sponsor must be notified in a timely manner, and a decision will be made to discontinue the study as appropriate. The investigator will ensure that the personal data of the subjects will be kept strictly confidential: all subject information and images will be identified by number rather than by name; identifiable information will not be disclosed outside the study team unless permission is obtained from the subject; all study members and the study sponsor will be asked to adhere to the principle of confidentiality; and all study files will be kept in locked filing cabinets for the sole use of the investigator; During and after completion of the study, government regulatory authorities or members of the ethics committee will be permitted to monitor subjects' personal data on a random basis as required; no personal information will be disclosed when the results of this study are published.

Signature of the researcher

I have read and agreed to the protocol proposed in this document, and agree with the relevant contents of the protocol, and will strictly abide by the laws and regulations of the People's Republic of China and relevant rules and regulations, fulfill my duties as a researcher and abide by the provisions of confidentiality in the course of the experiment.

Research Organization: The First Hospital Affiliated to Zhejiang University School of Medicine

Researcher's name: Huang He

Position of researcher: Principal investigator

Signed by the researcher:

Date of signature:

Protocol Summary

Name of study	Clinical study of CAR-T cell therapy for newly diagnosed Ph-positive B-cell acute lymphoblastic leukemia in adults
research phase	Phase 2
Research design	a single-arm, open-label, single-center design
Number of research centers	1
sample size	27 cases
indications	Ph-positive B-cell acute lymphoblastic leukemia in adults
research purpose	Main purpose:<br>Evaluating the efficacy of CAR-T cells for the treatment of newly diagnosed Ph-positive B-cell acute lymphoblastic leukemia in adults.<br>Secondary purpose:<br>Evaluating the safety of CAR-T cells for the treatment of newly diagnosed Ph-positive B-cell acute lymphoblastic leukemia in adults.<br>Exploratory purpose:<br>CAR-T cell expansion in vivo and B-cell clearance.
Primary endpoints	Complete molecular response (CMR) rate after CD19 CAR-T cell therapy
secondary endpoint	1) Complete molecular response (CMR) rate after CD22 CAR-T cell therapy<br>2) LFS, OS, and cumulative incidence of relapse (CIR);<br>3) Safety<br>4) Characterization of relapse (including expression of CD19, CD22 and mutations in the ABL1 gene)<br>**Exploratory Research Endpoints:**<br>CAR-T cell expansion in vivo and B-cell clearance.
Inclusion Criteria	**Inclusion criteria:**<br>1) Age ⩾ 18 years old;<br>2) Subjects with a diagnosis of B-cell acute lymphoblastic leukemia according to the 2016 edition of the WHO classification criteria for acute leukemia;<br>3) Subjects whose chromosomal and fusion gene analysis showed positivity for the Ph chromosome, BCR/ABL1 fusion gene;<br>4) Leukemia cells were CD19 and CD22 positive;<br>5) Patients with newly diagnosed B-ALL were not treated with standard chemotherapy regimens;<br>6) Serum total bilirubin ⩽ 51 mol/L, serum ALT and AST both ⩽ 3 times the upper limit of the normal range, blood creatinine ⩽ 176.8μ mol/L;<br>7) Echocardiography showed a left ventricular ejection fraction (LVEF) ≥50%;<br>8) Subjects had no active pulmonary infection and oxygen saturation ≥92% without oxygen;<br>9) The prognosis for survival is more than 3 months;<br>10) ECOG score 0-2;

	11) Subjects volunteered to participate in this trial and signed an informed consent form.
Exclusion criteria	Subjects with any of the following exclusion criteria were not eligible for enrollment in this trial:<br>1) Those with a history of epilepsy or other central nervous system disorders;<br>2) Those with a history of prolonged QT period or severe cardiac disease;<br>3) Women who are pregnant or breastfeeding (the safety of this therapy for the unborn child is not known);<br>4) Those with uncontrolled active infection;<br>5) Active hepatitis B or hepatitis C virus infection;<br>6) Those who have previously used any gene therapy product;<br>7) Those with insufficient amplification (<5-fold) in response to CD3/CD28 co-stimulatory signals;<br>8) Creatinine > 2.5 mg/dl or ALT / AST > 3 times the upper limit of the normal range or bilirubin > 2.0 mg/dl;<br>9) Those who suffer from other uncontrolled medical conditions that, in the opinion of the investigator, make them unsuitable for enrollment;<br>10) HIV-infected persons;<br>11) Any condition that, in the opinion of the investigator, may increase the risk to the subject or interfere with the results of the test.
Research termination criteria	1) the enrollment, treatment, and follow-up of the clinical study were successfully completed and reached the target number of cases; 2) the efficacy of the clinical trial far exceeded expectations; and 3) serious uncontrollable adverse events occurred in the clinical trial.
Visiting Program	1) CAR-T cells were infused on day 0;<br>2) Visit on the 4th, 7th, 9th, 11th, 14th, 21st, and 28th days after infusion (monitoring the condition - collecting blood specimens to determine CAR-T cell expansion, blood routine, blood biochemistry and others, and at the same time, it may be necessary to collect tissue samples, cerebrospinal fluid, pleural fluid, etc.);<br>3) Subjects were evaluated on a monthly basis for 6 months after infusion;<br>4) Subjects were evaluated at 3-monthly intervals for 2 years after infusion.
experimental procedure	**I. Signing the informed consent form**<br>A signed Informed Consent Form must be obtained from the subject before any study-related manipulations and assessments are performed.<br>**II. Screening period examination**<br>The screening period examination includes history, physical examination, vital signs, laboratory tests, and imaging.<br>**III. Induction chemotherapy**<br>Received dasatinib in combination with a two-week VP regimen (vindesine + dexamethasone).<br>**IV. Aphresis and lymphodepletion**

	Those achieved complete hematological remission proceed to aphresis and CAR-T cell preparations.<br>On days -4 to -2 prior to cell infusion (day 0), subjects will receive lymphodepletion with fludarabine + cyclophosphamide (FC regimen) as follows:<br>Fludarabine: 30 mg/m^2 /d, IV infusion, for three days from -4 days to -2 days;<br>Cyclophosphamide: 500 mg/m^2 /d, IV infusion, for two consecutive days from -3 days to -2 days.<br>On day -4, medicated lumbar puncture was given concurrently with the following regimen: methotrexate 10 mg, cytarabine 50 mg, dexamethasone 5 mg<br>**V. CAR-T Cell Therapy and Observations**<br>CAR-T cells were administered by intravenous infusion as a single dose. The duration of the infusion was 30 minutes.<br>**VI. Follow-up**<br>Follow-up visits are conducted at the time points specified in the protocol and assessments of test items, efficacy, and safety are completed.
quality management	Conducting studies, generating, recording and reporting data in strict accordance with the Code for Quality Management of Clinical Research in Cellular Immunotherapy and the corresponding regulatory requirements

catalogs

1 BACKGROUND TO THE STUDY **7**

2 PURPOSE OF THE CLINICAL STUDY **7**

3 OVERALL DESIGN **8**

3.1 RESEARCH DESIGN 8
3.2 PATIENT SELECTION 8
3.2.1 Inclusion criteria *8*
3.2.2 Exclusion criteria *8*
3.2.3 Criteria and procedures for subject termination from the study *8*
3.2.4 Time of enrollment *9*
3.2.5 Expected overall duration of clinical studies *9*
3.2.6 Number of subjects required for clinical studies *9*
3.3 STUDY ENDPOINTS 9
3.3.1 Main study endpoints *9*
3.3.2 Secondary research endpoints *9*

4 RESEARCH PROCESS **10**

4.1 INDUCTION CHEMOTHERAPY 10
4.2 APHRESIS 10
4.3 CAR-T CELL THERAPY AND MAINTENANCE 10
4.3.1 Assessment before lymphodepletion *10*
4.3.2 lymphodepletion *10*
4.3.3 CD19 CAR-T Cell Infusion *10*
4.3.4 CD22 CAR-T cell infusion *10*
4.3.5 Prevention of CNS leukemia *11*
4.3.6 Dasatinib maintenance *11*
4.4 RESEARCH DRUGS 11
4.4.1 Quality control of CAR-T cell preparation *11*
4.4.2 CAR-T Cell Infusion Environment *11*
4.4.3 Methods and precautions for administration of drugs *11*
4.5 RESEARCH PROCESS 12
4.5.1 Pharmacokinetic studies *12*
4.5.2 Research process *12*
4.6 ETHICS AND INFORMED CONSENT 22
4.6.1 Code of Practice for Quality Management of Drug Clinical Trials *22*
4.6.2 Responsibilities of the researcher *22*
4.6.3 Ethics Committee *22*
4.6.4 Related tables *22*
4.6.5 Ethical approval *22*
4.6.6 Informed consent *22*
4.6.7 Subject Privacy *23*
4.7 SUBJECT SCREENING 23

5 STATISTICAL CONSIDERATIONS **23**

5.1 STATISTICAL OVERVIEW 23
5.2 SAMPLE SIZE AND THE RATIONALE FOR ITS DETERMINATION 23
5.3 SIGNIFICANCE LEVEL AND CERTAINTY OF CLINICAL STUDIES 23

5.4 Statistical analysis of the population and statistical methods 24
5.4.1 Statistical analysis of populations *24*
5.4.2 Methods of statistical analysis *24*
5.5 Assessment and definition 24
5.6 Handling of missing values and outliers 24
5.7 Expected drop-out rate 24
6 DATA RECORDING AND MANAGEMENT 24
6.1 Case report form 24
6.2 Record-keeping 25
6.3 Source documents 25
7 CRITERIA AND PROCEDURES FOR TERMINATION OF CLINICAL STUDIES 25
8 EXPECTED PROGRESS OF THE STUDY 25
9 ASSESSMENT AND REPORTING OF ADVERSE EVENTS 26
9.1 Definition of Adverse Events 26
9.1.1 Adverse Events (AE) *26*
9.1.2 Serious Adverse Events (SAEs) *26*
9.1.3 Major adverse events *26*
9.2 Assessment of adverse events 28
9.2.1 Severity of adverse events *28*
9.3 Recording of adverse events 29
9.3.1 Recording of general adverse events *29*
9.3.2 Recording and reporting of serious adverse events *31*
9.4 Management and follow-up of adverse events 32
9.4.1 Handling of adverse events *32*
9.4.2 Costs of handling serious adverse events *32*
9.4.3 Follow-up of adverse events *32*
10 QUALITY CONTROL AND QUALITY ASSURANCE 33
10.1 Inspection visits to research centers 33
10.2 Protocol Violations 33
10.3 Quality assurance audits 33
Appendix Treatment of Cytokine Release Syndrome (CRS) 34

1 Background to the study

Acute lymphoblastic leukemia (ALL) is a malignant clonal disease of lymphoid precursor cells, in which abnormal primitive and naïve lymphocytes (leukemic cells) proliferate in the bone marrow and inhibit normal hematopoiesis, and may extensively infiltrate various organs such as liver, spleen and lymph nodes. The incidence rate of ALL in China is 1.3 per 100,000, which is similar to that in other Asian countries and lower than that in Europe and the U.S. The age distribution of ALL shows a bimodal peak, with the incidence rate reaching a peak in childhood and around the age of 60 years.ALL is the most common tumor in children. With current international standard chemotherapy regimens, childhood ALL has become a curable disease with a five-year overall survival (OS) rate of 89%, compared to 61% for adolescent and young adult ALL. In contrast, the long-term OS for adult ALL is approximately 20% to 40%, and most adults will eventually relapse due to chemotherapy resistance. The prognosis for relapsed or refractory ALL is extremely poor, with a five-year OS of only 10%. One of the major reasons why adult ALL is less effective than pediatric ALL is the significantly higher proportion of high-risk genetic abnormalities in adult ALL, with Ph chromosome and BCR/ABL1 fusion gene positivity being the most important ones.

In recent years, there have been significant advances in immunotherapy, many of which target surface antigens on B lymphocytes such as CD19, CD20, and CD22, including monoclonal antibodies (mAb), antibody-drug conjugates, bispecific T cell engager (BiTE), and chimeric antigen receptor T cells (CAR-T). Immunotherapy has made breakthroughs in the treatment of relapsed refractory B-ALL, so that immunotherapy such as Blinatumomab in combination with the tyrosine kinase inhibitor (TKI) dasatinib has been studied in the first-line treatment of Ph-positive ALL with significant efficacy. CAR-T cell therapy has also shown breakthroughs in refractory relapsed B-ALL, with some patients achieving sustained remission and long-term survival. It is worth exploring whether CAR-T can be used in combination with dasatinib for the first-line treatment of newly diagnosed Ph-positive ALL to improve the prognosis of the disease. Therefore, we propose to apply for a clinical study on the efficacy and safety of CD19 CAR-T cells combined with dasatinib for the treatment of adult newly diagnosed Ph-positive B-ALL. To minimize the relapse rate, CD19 CAR-T cell therapy will be followed by sequential CD22 CAR-T cell therapy. This sequential strategy was shown to significantly reduce the relapse rate in refractory or relapsed B-ALL.

2 Purpose of the clinical study

1) To evaluate the efficacy of CD19 CAR-T in combination with dasatinib for the treatment of newly diagnosed Ph-positive B-cell acute lymphoblastic leukemia in adult.

2) To evaluate the safety of CD19 CAR-T in combination with dasatinib for the treatment of newly diagnosed Ph-positive B-cell acute lymphoblastic leukemia in adult.

3 Overall design

3.1 Research design

This study was designed as a prospective, open-label, single-center study. It aims to evaluate the efficacy and safety of CD19 CAR-T cells in combination with dasatinib for the treatment of newly diagnosed Ph-positive B-cell acute lymphoblastic leukemia in adult.

3.2 Patient selection

3.2.1 Inclusion criteria

1) Age ≥ 18 years old;
2) Subjects with a diagnosis of B-cell acute lymphoblastic leukemia according to the 2016 edition of the WHO classification criteria for acute leukemia;
3) Subjects whose chromosomal and fusion gene analysis showed positivity for the Ph chromosome, BCR/ABL1 fusion gene;
4) Leukemia cells were CD19 and CD22 positive;
5) Patients with newly diagnosed B-ALL were not treated with standard chemotherapy regimens;
6) Serum total bilirubin ≤ 51 mol/L, serum ALT and AST both ≤ 3 times the upper limit of the normal range, blood creatinine ≤ 176.8μ mol/L;
7) Echocardiography showed a left ventricular ejection fraction (LVEF) ≥50%;
8) Subjects had no active pulmonary infection and oxygen saturation ≥92% without oxygen;
9) The prognosis for survival is more than 3 months;
10) ECOG score 0-2;
11) Subjects volunteered to participate in this trial and signed an informed consent form.

3.2.2 Exclusion criteria

Subjects with any of the following exclusion criteria were not eligible for enrollment in this trial:

1) Those with a history of epilepsy or other central nervous system disorders;

2) Those with a history of prolonged QT period or severe cardiac disease;

3) Women who are pregnant or breastfeeding (the safety of this therapy for the unborn child is not known);

4) Those with uncontrolled active infection;

5) Active hepatitis B or hepatitis C virus infection;

6) Those who have previously used any gene therapy product;

7) Those with insufficient amplification (<5-fold) in response to CD3 / CD28 co-stimulatory signals;

8) Creatinine > 2.5 mg/dl or ALT / AST > 3 times normal or bilirubin > 2.0 mg/dl;

9) Those who suffer from other uncontrolled medical conditions that, in the opinion of the investigator, make them unsuitable for enrollment;

10) HIV-infected persons;

11) Any condition that, in the opinion of the investigator, may increase the risk to the subject or interfere with the results of the test.

3.2.3 Criteria and procedures for subject termination from the study

1) Subjects who do not complete the study protocol are considered to have terminated the study early. Reasons for termination (e.g., voluntary withdrawal, development of a serious adverse reaction, death) must be documented

on a Case Report Form (CRF) and maintained for a specified period of time as required by GCP. Possible reasons for early termination and withdrawal include:

A. Adverse Events. An adverse event occurs in a subject and the investigator believes that discontinuing the trial is a medical decision based on the greatest benefit to the subject;

B. Lack of efficacy. The investigator determines that the subject has not benefited from the trial treatment and that continued participation in the study may place the subject at unpredictable risk;

C. Significant Protocol Violation. After CAR-T cell infusion, the subject is found to not meet the inclusion criteria or to be in noncompliance with the requirements of the protocol;

D. Subject refuses to continue treatment or observation and voluntarily withdraws;

E. Subject Missed Visit. The subject did not return to the hospital on time for the follow-up visit and the investigator's attempts to contact the subject failed. Efforts to contact the subject must be documented;

F. Subject death. Record and report according to SAE;

G. Pregnancy Events. Once a subject is found to be pregnant, the subject must be immediately withdrawn from the study and documented and reported in accordance with the SAE;

H. Study Termination. The Collaborator, IRB, IEC, or regulatory agency requests that the study be terminated;

I. Subjects who may not be able to complete this study for other reasons or who, in the judgment of the investigator, are not suitable for participation.

2) Withdraw procedure

The investigator, in order to ensure the best interests of the subjects, may discontinue subjects who are not suitable for the study protocol based on a clinical diagnosis and subjects with poor compliance. Subjects may withdraw informed consent at any time during the study without discrimination.

3.2.4 Time of enrollment

From the date of ethical approval

3.2.5 Expected overall duration of clinical studies

3 years

3.2.6 Number of subjects required for clinical studies

27 adult patients with newly diagnosed Ph-positive B-cell acute lymphoblastic leukemia

3.3 Study endpoints

3.3.1 Main study endpoints

Complete molecular response (CMR) rate after CD19 CAR-T cell therapy

3.3.2 Secondary research endpoints

1) Complete molecular response (CMR) after CD22 CAR-T cell therapy
2) LFS, OS, and cumulative relapse rate (CIR);
3) Safety
4) Characterization of relapse (including expression of CD19, CD22 and mutations in the ABL1 gene).

3.3.3 Exploratory study endpoints:

CAR-T cell expansion in vivo and B-cell clearance.

4 Research Process

4.1 Induction chemotherapy

Patients received a pre-treatment of glucocorticoids (dexamethasone 10 mg intravenously once daily) for 5-7 days. For patients with white blood cell counts above 30,000/μL, cyclophosphamide 300 mg per day is also given intravenously to reduce leukemia burden, combined with leukapheresis if necessary. necessary. This was followed by a two-week induction regimen of vindesine and glucocorticoids (vindesine 4 mg intravenously on days 1 and 8; dexamethasone 10 mg intravenously on days 1-11 and 5 mg intravenously on days 12-14). Dasatinib was added to the induction regimen at a dose of 100 mg once daily.

4.2 Aphresis

Patients who have completed induction chemotherapy and achieved complete hematologic remission will be subjected to aphresis. CAR-T cells are produced by Shanghai Yako Biotechnology Co.

4.3 CAR-T Cell Therapy and Maintenance

4.3.1 Assessment before lymphodepletion

Before lymphodepletion, investigators should evaluate patients' infections and immune status. For patients with uncontrolled systemic infections, the relevant procedures after enrollment should be suspended. The timing of lymphodepletion and CAR-T cell infusion should be reassessed.

4.3.2 lymphodepletion

Subjects receive FC (fludarabine + cyclophosphamide) regimen for lymphodepletion as follows:

Fludarabine 30 mg/m^2, was administered intravenously for 3 consecutive days (days -4 to -2);

Cyclophosphamide 500 mg/m^2, was administered intravenously for 2 consecutive days (days -3 to -2);

Chemotherapy-related formulas:

Body surface area BSA (m^2) = 0.0061 x height (cm) + 0.0128 x weight (kg) - 0.1529;

Fludarabine (mg) = BSA x 30 mg/m^2 ;

Cyclophosphamide (mg) = BSA x 500 mg/m^2 .

In this case, the subject's body surface area (BSA) was calculated based on the most recent height/weight prior to lymphodepletion, and it was preferred to calculate the BSA using the height/weight values measured on the day prior to lymphodepletion.

4.3.3 CD19 CAR-T Cell Infusion

CAR-T cell therapy was infused on day 0. Under special circumstances, investigators may opportunistically infuse CAR-T cells based on patient's condition. The target dose of CAR-T cells was 2×10^6 CAR+ T cells/kg (dose range ± 20%), and the lowest acceptable dose was 1×10^6 CAR+ T cells/ kg.

4.3.4 CD22 CAR-T cell infusion

The timing of CD22 CAR-T cell therapy includes failure to achieve complete molecular response or normal B-cell reappearance after CD19 CAR-T cell therapy. The process of CD22 CAR-T cell therapy is the same as CD19 CAR-T cell therapy.

4.3.5 Prevention of CNS leukemia

Central nervous system (CNS) prophylaxis with medicated lumbar punctures (methotrexate 10 mg, cytarabine 50 mg, and dexamethasone 5 mg) was given twice in total, each time on the first day of lymphodepleting chemotherapy.

4.3.6 Dasatinib maintenance

Dasatinib discontinued during CAR-T cell therapy and restarted after recovery of neutropenia and thrombocytopenia to grade 1. All patients received single-agent dasatinib maintenance (at a dose of 100mg once daily) after sequential CAR-T cell therapies. Dasatinib was maintained for a minimum of 2 years, with continuation after 2 years at the discretion of the investigator.

4.4 Research drugs

4.4.1 Quality control of CAR-T cell preparation

①Quality control of prepared materials

All materials used in the preparation process should comply with the principle of aseptic safety, and establish a perfect registration and monitoring system for the preparation materials, and set up corresponding safety and utility testing programs, such as bacterial, fungal, mycoplasma and exogenous viral contamination testing, and purity, potency and other tests related to cell activation and proliferation.

② Quality control of plasmid vectors and viral vectors

Quality control of plasmid vectors: through the detection of plasmid sequence, copy number and restriction zymography to prove that the plasmid in the strain contains the target element and the corresponding copy number; through the detection of plasmid retention rate to verify the stability of the strain and to limit the use of the strain to the level of transmissibility. The control of plasmid purity includes two aspects, one is the quality of the plasmid itself. On the other hand, it is the control of impurities in the process, such as host bacterial protein residues, host bacterial DNA residues, and other components added in the process that need to be controlled.

Quality control of viral vectors: Viral vectors need to be evaluated for vector titer, and the ability to transduce cells is usually adopted as the titer of viral vectors. In addition, each batch of viral vectors is tested for exogenous factor contamination, specifically bacterial, fungal, mycoplasma and exogenous viral factor contamination.

③ Quality control of CAR-T cell production: after the preparation of CAR-T cells is completed, it is necessary to test the number of cells, cell viability, cell phenotype, CAR positivity test, biological efficacy test, and safety test.

4.4.2 CAR-T Cell Infusion Environment

The environment for CAR-T cell infusion in this study is sufficient according to the routine clinical transfusion environment requirements.

4.4.3 Methods and precautions for administration of drugs

The study drug is a CAR-T cell preparation for intravenous infusion. Refer to the following requirements for specific use (as an example of frozen product):

① Thaw CAR-T cell preparations using saline at 37° C until there is no visible ice in the infusion bag.

② Check whether there are any visible cell clumps in the contents of the thawed infusion bag, if they still exist, the contents of the bag should be gently mixed, and small clumps can be dispersed by gently flicking with the hand. If clumps are not dispersed, the infusion bag is damaged, leaking or other abnormalities occur, the product should not be infused, and the investigator should promptly contact Shanghai Yakel Biotechnology Co.

③ Once the CAR-T has been thawed and is at room temperature (20° C to 25° C), the infusion should be completed within 30 minutes. Do not wash or resuspend CAR-T cells with fresh solution prior to infusion.

④ The recommended drip rate for intravenous infusion of CAR-T is 20 to 40 drops per minute, and the investigator may adjust the drip rate appropriately according to the condition of the subject. Care should be taken during the infusion process:

Do not use a leukocyte filter;

Use saline to lubricate the tubing prior to infusion;

4.5 Research process

4.5.1 Pharmacokinetic studies

In order to fully investigate the proliferation and persistence of CAR-T cells in subjects, this trial will continue to conduct pharmacokinetic studies, the main observations are the peak concentration, time to peak, and T-cell persistence time, and the monitoring methods are as follows:

1) Flow cytometry

2) Quantitative real-time polymerase chain reaction

In addition to the above analytical methods, it may be possible to dynamically monitor changes in CAR-T cells in vivo by other validated assays, if necessary.

Monitoring of CAR-T begins 1 day before cell infusion and ends at the end of the therapeutic observation period, during which pharmacokinetic index observations are required at each visit; subjects should also be continuously monitored after entering the survival follow-up period, with the endpoint of pharmacokinetic monitoring being the inability to detect CAR-T in vivo, i.e., two consecutive negative monitoring results.

4.5.2 Research process

In this study, the screening period was within 14 days, the induction period was about 30 days, the cell preparation and lymphodepletion period was about 15 days, the CAR-T cell therapy observation period was 29 days, and the CAR-T cell therapy follow-up period was 62 days, totaling about 150 days for the above five phases.

Ninety days after the infusion of CAR-T, patients enter the survival follow-up period, with near-term safety follow-up until CAR-T cells are undetectable in vivo or 2 years after infusion, whichever is longer; because of the potential tumorigenicity that may be present in CAR-T cellular preparations, long-term safety follow-up should be lifelong or last for at least 15 years for tumorigenicity in subjects.

1) Screening period (within 14 days prior to enrollment, V1)

After signing the informed consent form, the following examinations and assessments were completed within 14 days, and enrollment was determined:

Signed informed consent;

Demographic information: sex, date of birth, ethnicity, occupation, marriage;

Medical history confirmation, including: history of B-ALL and related treatments; history of other malignancies; history of other medical conditions (including, but not limited to: cardiopulmonary disease, psychiatric disorders, active autoimmune disease, active infectious disease, etc.);

Vital signs: temperature, respiration, heart rate (or pulse), blood pressure, height, weight;

Physical examination: general condition, skin and mucous membranes, lymph nodes, head, neck, chest, heart, abdomen, genitourinary system, spine extremities, nervous system, mental status and others; height and weight;

Blood count: white blood cell count (WBC), red blood cell count (RBC), hemoglobin (Hb), platelet count (PLT), absolute neutrophil count (Neut#), absolute lymphocyte count (Lymph#), absolute monocyte count (Mono#);

Coagulation: prothrombin time (PT), international normalized ratio (INR), activated partial thromboplastin time (APTT);

Blood biochemistry:

Electrolytes: potassium (K), sodium (Na), chlorine (Cl), calcium (Ga), phosphorus (P), magnesium (Mg);

Liver function: alanine aminotransferase (ALT), (AST), gamma-glutamyltransferase (GGT), alkaline phosphatase (ALP), total protein (TP), albumin (ALB), globulin (GLOB), total bilirubin (TBIL), and lactate dehydrogenase (LDH);

Renal function: urea (Urea), blood creatinine (SCr), uric acid (UA), glomerular filtration rate (GFR) or creatinine clearance Ccr;

Lipids: total cholesterol (TC), triglycerides (TG), high-density lipoprotein (HDL-C), low-density lipoprotein (LDL-C);

Glucose (GLU);

Echocardiography: left ventricular ejection fraction (LVEF);

Virology examination: including hepatitis B virus (HBV), hepatitis C virus (HCV), human immunodeficiency virus (HIV), treponema pallidum (TP), EB virus (EBV), cytomegalovirus (CMV); those who are not known by antigen-antibody screening method are confirmed by the nucleic acid method, of which those who are positive in virology antigen-antibody screening for HCV, EBV, CMV, need to have additional quantitative nucleic acid method examination, i.e. HCV-RNA, EBV-DNA, CMV-DNA; HBV-DNA is required for those who are positive for HBsAg and HBeAg; the above virology programs are acceptable for the results of the examination within one month prior to enrollment, except for HIV;

ECOG score;

Pregnancy testing: All women of gestational age (including tubal ligation) during the screening period are required to undergo a pregnancy test;

Bone marrow examination: bone marrow smear observation, leukemia immunophenotyping (CD19, CD22 expression);

Peripheral blood smear observation;

Imaging: performed according to study needs, or CT and/or MRI or PET-CT;

Rheumatologic tests: qualitative antinuclear antibody (ANA) tests;

Urine Routine:

Urine dry chemistry: urobilinogen (UBG), bilirubin (BIL), ketone bodies (KET), occult blood (ERY), urine protein (PRO), glucose (GLU), pH, vitamin C (VC), specific gravity of urine (SG);

Urine sediment microscopy: red blood cells (RBC), white blood cells (WBC);

12-lead electrocardiogram;

Immunoglobulins: complement C3, complement C4, immunoglobulin A (IgA), immunoglobulin G (IgG), immunoglobulin M (IgM);

Ferritin;

C-reactive protein;

Calcitoninogen (PCT);

Cardiac enzymes: B-type natriuretic peptide (BNP) or B-type natriuretic peptide prepeptide (NT-proBNP);

Lymphocyte subsets: CD3, CD4, CD8, CD16, CD45, CD56;

Expected Survival Assessment;

Adverse events;

Combined medication.

Unless otherwise requested, the above examinations and contents are the same as those described below.

Based on the above examination results, the inclusion/exclusion criteria were checked one by one, and the investigator confirmed whether the screened patients were enrolled or not; during the screening process, if there were already sufficiently strong materials to prove that the patients did not meet the requirements for enrollment, the examination items that were not carried out could not be carried out.

2) Induction chemotherapy period (approximately 30 days after enrollment, V2)

First week of induction chemotherapy

Daily: vital signs; physical examination; complete blood counts

Every two days: blood biochemistry

Twice weekly: coagulation; cardiac enzymes; C-reactive protein

Second week of induction chemotherapy

Daily: vital signs; physical examination

Every two days: complete blood counts

Twice weekly: blood biochemistry; coagulation; cardiac enzymes; C-reactive protein

Third to fourth week of induction chemotherapy

Daily: vital signs; physical examination

Twice weekly: complete blood counts; blood biochemistry; cardiac enzymes; C-reactive protein

Weekly: Coagulation

D28: Bone marrow examination

3) Cell preparation and Lymphodepletion period, V3

Cell preparation

After receiving induction chemotherapy, subjects who achieve CHR will proceed to aphresis, and the CAR-T cells will be prepared by Shanghai Arco Biotechnology Co.

Lymphodepletion period

The following tests were performed prior to lymphodepletion, including:

Vital signs; physical examination; CBC; coagulation; blood biochemistry; echocardiography; viral workup; imaging; cerebrospinal fluid examination (D-4); routine urine; immunoglobulins; ferritin; C-reactive protein; calcitonin proteins; cardiac enzymes; cytokines; lymphocyte subsets; T-cell subsets; CAR copies; Q-PCR; adverse events; comorbid medications.

Among them, cytokines include IL-2, IL-6, etc. (below), and T cell subsets include CAR, CD3, CD4, CD8, etc. (below).

3) Treatment Observation Period (D0~D28)

The day when the subjects received the infusion of CAR-T cells was recorded as D0, and the following 28 days were followed up for the observation period of the treatment, and the appropriate items were examined to evaluate the safety of the treatment.

D0(V4)

Vital signs; physical examination; cytokines; lymphocyte subsets; T-cell subsets; CAR copies; Q-PCR; adverse events; comorbidities.

In this case, the physical examination, except for height and weight, is only required to record significant changes from baseline (below).

d4 (v5), d7 (v6), d9 (v7), d11 (v8)

Vital signs; physical examination; CBC; coagulation; blood biochemistry; routine urine; immunoglobulins; ferritin; C-reactive protein; calcitoninogen; cardiac enzymes; cytokines; lymphocyte subsets; T-cell subsets; CAR copies; adverse events; coadministered medications.

D14 (V9), D21±1 (V10)

Vital signs; physical examination; CBC; coagulation; blood biochemistry; peripheral blood smear observation; routine urine; immunoglobulins; ferritin; C-reactive protein; calcitoninogen; cardiac enzymes; cytokines; lymphocyte subsets; T-cell subsets; CAR copies; Q-PCR; adverse events; comorbid medications.

D28±1 (V11)

The primary endpoint of this study, assessing the effectiveness of the study drug.

The following inspections were primarily completed:

Vital signs; physical examination; CBC; coagulation; blood biochemistry; bone marrow examination; peripheral blood smear observation; routine urine; 12-lead electrocardiogram; immunoglobulin; ferritin; C-reactive protein; procalcitonin; cardiac enzymes; cytokines; lymphocyte subpopulations; T-cell subsets; CAR copies; Q-PCR; adverse events; comorbid medications.

4) Treatment follow-up period (D29-D90)

After completion of the treatment observation period, the investigator is still required to follow up on the effectiveness and safety of the treatment (62 days in total) and complete the appropriate examinations.

D56±3 (V12)

Vital signs; physical examination; CBC; coagulation; blood biochemistry; bone marrow examination; peripheral blood smear observation; routine urine; immunoglobulins; ferritin; C-reactive protein; calcitoninogen; cardiac enzymes; cytokines; lymphocyte subsets; T-cell subsets; CAR copies; Q-PCR; adverse events; comorbid medications.

D90±3 (V13)

Vital signs; physical examination; CBC; coagulation; blood biochemistry; echocardiography; ECOG score; bone marrow examination; peripheral blood smear observation; routine urinalysis; 12-lead electrocardiogram; immunoglobulins; ferritin; C-reactive protein; procalcitonin; cardiac enzymes; cytokines; lymphocyte subpopulations; T-cell subsets; CAR copies; Q-PCR; adverse events; comorbid medications.

Women of gestational age are also required to undergo: a pregnancy test.

Prior to the end of the treatment follow-up period, in the event of early withdrawal, subjects should endeavor to complete the pre-withdrawal visit and assessment. Patients who withdraw early from the study or terminate the study should complete the early withdrawal visit as soon as possible (within 1 week of withdrawal), which is required to be performed according to V17, and should be completed and evaluated prior to receiving new treatment.

5) Survival follow-up period (after D90)

At the end of the treatment follow-up period, subjects entered the survival follow-up period, and depending on the persistence of the CAR-T cells in vivo, the investigator instructed him or her to follow up at the appropriate time to improve pharmacokinetic monitoring, which should be done at least once every three months until two consecutive test results are negative or death occurs.

In addition, for patients in the survival follow-up period, the investigator conducts a follow-up visit every three months, which can be done by on-site visit or telephone visit, in order to understand their physical and living conditions and collect data on the follow-up treatment, and record the details of the follow-up visit as exhaustively

as possible. Near-term survival follow-up lasts for two years or CAR-T test is negative, whichever is longer; long-term safety follow-up should be lifelong or last for 15 years for the tumorigenicity of the subject.

The following information should also be obtained throughout the survival follow-up period:

1) Survival time assessment, including date of assessment;

2) If a death occurs, the date of death shall be recorded;

3) All new cancer cases.

Survival follow-up should also continue with study-related safety follow-up until the follow-up endpoint for adverse events is reached.

Throughout the study period, the investigator may increase or decrease the number of examinations or visit points according to the clinical reality, but the reasons for the increase or decrease should be recorded as far as possible. If the same test is performed more than once at the same visit for study purposes, the results of the first test performed will be the primary study data, and the remaining tests should be recorded.

Table 1 Clinical study flowchart

**study period***	**screening period[a]**	**induction period**			**Cell preparation and lymphodepletion period**	**CAR-T treatment observation period (D)[b]**								**CAR-T treatment follow-up period (D)[b]**		**Survival follow-up period (D)[c]**
**Diagnostic/Evaluation items**	**Within 14 days prior to enrollment**	**Week 1**	**Week 2**	**Weeks 3-4**	**About 15 days**	**0**	**4**	**7**	**9**	**11**	**14**	**21±1**	**28±1**	**56±3**	**90±3**	**After 90**
**visitation point**	**V1**	**V2**			**V3**	**V4**	**V5**	**V6**	**V7**	**V8**	**V9**	**V10**	**V11**	**V12**	**V13**	**---**
informed consent	●															
Demographic information[1]	●															
medical history[2]	●															
vital signs[3]	●	Daily	Daily	Daily	●	●	●	●	●	●	●	●	●	●	●	
Physical examination[4]	●	Daily	Daily	Daily	●	●	●	●	●	●	●	●	●	●	●	
CBC[5]	●	Daily	Every other day	Twice a week	●		●	●	●	●	●	●	●	●	●	
coagulation[6]	●	Twice a week	Twice a week	Once a week	●		●	●	●	●	●	●	●	●	●	
blood biochemistry[7]	●	Every other day	Twice a week	Twice a week	●		●	●	●	●	●	●	●	●	●	
echocardiography[8]	●				●										●	
Virology[9]	●				●											
ECOG score	●														●	

Pregnancy test[10]	●														●	
Histopathological examination[11]	●														●	
Bone marrow examination[12]	●			●D28									●	●	●	
Cerebrospinal fluid examination[13]					●D-4											
Peripheral blood smear	●			●D28									●	●	●	
Imaging[14]	●				●										●	
Rheumatology[15]	●															
urine routine[16]	●				●		●	●	●	●	●	●	●	●	●	
12-lead electrocardiogram	●				●								●		●	
immunoglobulin[17]	●				●		●	●	●	●	●	●	●	●	●	
ferritin	●				●		●	●	●	●	●	●	●	●	●	
C-reactive protein	●	Twice a week	Twice a week	Twice a week	●		●	●	●	●	●	●	●	●	●	
calcitonin	●				●		●	●	●	●	●	●	●	●	●	
cardiac myosin[18]	●	Twice a week	Twice a week	Twice a week	●		●	●	●	●	●	●	●	●	●	
cytokine[19]					●	●	●	●	●	●	●	●	●	●	●	
lymphocyte subset[20]	●				●	●	●	●	●	●	●	●	●	●	●	

Assessment of expected survival	●															
Verification of entry into the study	●															
Aphresis and preparation					●											
lymphodepletion					●											
CAR Copy Q-PCR[21]					●	●	●	●	●	●	●	●	●	●	●	●
T-cell subset[22]					●	●	●	●	●	●	●	●	●	●	●	●
CAR-T infusion[23]						●										
adverse event [I]	●	●	●	●	●	●	●	●	●	●	●	●	●	●	●	●
Combined treatment/medication [II]	●	●	●	●	●	●	●	●	●	●	●	●	●	●	●	●

Note 1:

a) Screening Provisions: 1) If a patient has sufficiently strong documentation during screening that he or she meets the enrollment requirements, the remaining assessment items in this study may not be performed;

b) Subjects who withdraw early or terminate from the study during the study should try to complete the early exit visit as soon as possible, within one week;

c) For subjects in the survival follow-up period, based on the persistence of CAR-T cells in the patient's body, the investigator instructed them to have timely follow-up visits to improve pharmacokinetic monitoring, which should be at least once every three months until two consecutive test results show negative results or 2 years after infusion; thereafter, follow-up visits should still be conducted once every three months, which can be done through off-site visits such as telephone calls, in order to observe their survival status.

Note 2:

1. Demographic information: sex, date of birth, ethnicity, occupation, marriage;

2. Medical history: 1) history of B-cell acute lymphoblastic leukemia; 2) history of other malignancies; 3) history of other medical conditions (including, but not limited to: cardiopulmonary disease, psychiatric disorders, active autoimmune disease, active infectious disease, etc.);

3. Vital signs: respiration, heart rate (or pulse), temperature, blood pressure; blood oxygen saturation observations should also be increased before and after cytosine infusion;

4. Physical examination: general condition, skin and mucous membranes, lymph nodes, head, neck, chest, heart, abdomen, genitourinary system, spine and limbs, nervous system, mental status and others are required for the screening period and D-1; thereafter, the physical examination is required to record only the obvious changes; height and weight are measured according to the requirements of the visit;

5. Complete Blood count: white blood cell count, red blood cell count, hemoglobin, platelet count, absolute neutrophil count, absolute lymphocyte count, absolute monocyte count;

6. Coagulation: prothrombin time, international normalized ratio, activated partial thromboplastin time;

7. Blood biochemistry: 1) Electrolytes: potassium, sodium, chloride, calcium, phosphorus, magnesium; 2) Liver function: alanine aminotransferase, glutamine aminotransferase, gamma-glutamyltransferase, alkaline phosphatase, total protein, albumin, globulin, total bilirubin, lactic acid dehydrogenase; 3) Renal function: urea, blood creatinine, uric acid, glomerular filtration rate GFR, or creatinine clearance Ccr; 4) Total cholesterol, triglyceride, high density lipoprotein, low-density lipoprotein; 5) blood glucose;

8. Echocardiography: left ventricular ejection fraction LVEF;

9. Virological examination: HBV, HCV, HIV, TP, EBV, CMV virological antibody examination; among them, those who are positive in HCV, EBV, CMV virological antibody screening need to be additionally examined by quantitative examination of nucleic acid method, i.e. HCV-RNA, EBV-DNA, CMV-DNA; Hepatitis B two-half pairs (HBsAg, HBsAb, HBeAg, HBeAb, HBcAb) of HBsAg and HBeAg positive, need to be tested by nucleic acid method, i.e., HBV-DNA; the results of the above screenings within 1 month prior to screening are acceptable, except for HIV;

10. Pregnancy testing: pregnancy testing is required for all women of gestational age (including tubal ligation); pregnancy testing should be performed at the end of the study/early exit termination visit;

11. Histopathological examination: tumor tissue CD19 positive expression rate test, of which, flow cytometry results or pathological tissue wax block results within 2 months are acceptable in the screening period;

12. Bone marrow examination: bone marrow MRD examination and/or bone marrow smear;

13. Cerebrospinal fluid examination: Cerebrospinal fluid MRD is required for leukemia invading the central nervous system;

14. Imaging: performed according to study needs, or CT and/or MRI or PET-CT;

15. Rheumatologic tests: qualitative antinuclear antibody (ANA) tests;

16. Urine routine: 1) urine dry chemistry: urinary bilogen, bilirubin, ketone bodies, occult blood, urinary protein, urinary glucose, urinary pH, urine specific gravity, vitamin C; 2) urine sediment microscopy: red blood cells, white blood cells;

17. Immunoglobulins: complement C3, complement C4, immunoglobulin A, immunoglobulin G, immunoglobulin M;

18. Cardiac enzymes: B-type natriuretic peptide or B-type natriuretic peptide prepeptide;

19. Cytokines: IL-2, IL-6, etc., and the investigator may add essential factor tests as needed;

20. Lymphocyte subsets: CD3, CD4, CD8, CD16, CD45, CD56, etc;

21. CAR copy, Q-PCR: can be tested according to the Clinical Research Center;

22. T-cell subsets: CAR, CD3, CD4, CD8, etc., can be sent to a third party for testing, depending on the clinical research center testing;

23. CAR-T infusion: single infusion

Each of the above tests may be increased or decreased as necessary by the investigator depending on the patient's condition, but the reason for the increase or decrease should be recorded.

If the same inspection is carried out several times at the same site, the results of the first inspection will be the main data of the study, and the results of the other inspections should also be recorded.

Note 3:

i. Requirements for adverse events: 1) Starting with the Screening Period, record all AEs and report SAEs; 2) Subjects withdrawing from the trial should continue to be followed up for adverse event outcomes as far as possible up to the AE follow-up endpoints; and 3) During the Survival Follow-up Period, record AEs related to this study and report SAEs related to this study;

ii. Requirements for comorbid treatments/medications: 1) Record all comorbid medications and treatments from the start of screening through the treatment follow-up period; 2) Record study-related comorbid medications and treatments when the subject withdraws or enters the survival follow-up period and record the most recent antitumor intervention, if other antitumor interventions have occurred.

4.6 Ethics and Informed Consent

4.6.1 Code of Practice for Quality Management of Drug Clinical Trials

The operations outlined in this Clinical Trial Protocol regarding the conduct, evaluation and documentation of this study are intended to ensure that its authorized representatives and investigators comply with the basic ethical requirements outlined in the GCP regulations and the Declaration of Helsinki. Approval from the Ethics Committee should be obtained prior to the commencement of this trial. The investigator should comply with applicable regulatory requirements of national, provincial and local regulatory authorities in all aspects of the execution of the trial.

4.6.2 Responsibilities of the researcher

See GCP and local regulatory requirements for investigator responsibilities. The investigator should be authorized to perform the work involved. The investigator should ensure that all personnel assisting in the execution of the study are fully informed about the study protocol and its amendments, the study treatment process, and the duties and functions associated with the study. The investigator should maintain a list of assistant investigators and other personnel who are appropriately qualified and who have assumed a significant division of labor related to the study.

The investigator is responsible for maintaining a record of all subjects who have signed the informed consent form and have been screened into the study. For subjects who fail screening, the reason for the screening failure must be documented in the subject's source file.

The investigator or his/her designated staff member must be present at the monitoring visit to allow access to data, resolution of challenges, and direct access to subject records (e.g., medical records, clinic record forms, hospital record forms, and study-related record forms) for verification of raw data. Investigators must ensure that CRFs and data challenges are completed in a timely and accurate manner.

4.6.3 Ethics Committee

The trial should not be started until the trial protocol and information such as the informed consent form have been approved by the Ethics Committee (EC). The EC should be composed in accordance with the requirements of the NMPA and carry out all the duties specified.

4.6.4 Related tables

Forms involved in clinical trials are subject to EC requirements.

4.6.5 Ethical approval

Prior to the commencement of the trial, the investigator was required to submit the study protocol, informed consent, CRF, approvals from the relevant authorities, and any advertisements for recruitment of subjects to the Ethics Committee of the Clinical Research Group Leader's Unit or to an independent Ethics Committee of another Clinical Research Unit for approval. Investigators were allowed to enroll cases only after receiving the ethical approval. If the trial protocol needs to be changed later, it must be submitted to the EC for review. The trial can only continue after the changes in the protocol and the revised informed consent form have been reviewed and approved by the EC.

4.6.6 Informed consent

The informed consent form must be submitted to the appropriate ethics committee for approval and the benefits and risks of the trial must be fully explained to each potential subject before they participate in any activity related to the trial. After explaining the basics of the trial and being satisfied that each prospective subject understands the

purpose of the trial, the investigator should ask each prospective subject to sign and date the informed consent form. Two copies of the informed consent form shall be kept by the investigator and one by the subject. If important new information concerning the trial drug is discovered, the informed consent form must be revised in writing and sent to the Ethics Committee for approval before obtaining the consent of the subjects again. All versions of ICF approved by the Ethics Committee had to be signed by the current participants and used for future recruitment of subjects.

4.6.7 Subject Privacy

Investigators and collaborators are responsible for respecting the privacy of trial subjects and for respecting the confidentiality of records.

4.7 Subject Screening

Subjects will sign an informed consent form prior to enrollment in this study. The investigator will explain the nature, purpose and risks of the study and provide subjects with a copy of the informed consent form. Subjects will be given adequate time to consider the implications of the study before deciding whether or not to participate. Any changes to the informed consent form will require ethics notification and approval prior to subject enrollment.

Firstly, subjects need to go through the informed consent process and sign the informed consent form, and then according to the pre-established inclusion and exclusion criteria of this study, the subjects who meet the criteria are screened from the target population in order to be regarded as the subject can be enrolled in this study.

General examination, medical history, laboratory tests, electrocardiogram and angiographic data from the subject's home or outside hospital within one week prior to signing the informed consent form can be collected as baseline values. Adverse events, concomitant medications need to be collected after signing the informed consent.

5 Statistical considerations

5.1 Statistical overview

In order to comply with the clinical trial protocol, follow the "Code of Practice for the Administration of Clinical Trials of Pharmaceuticals" of the People's Republic of China, smoothly implement the trial, generate, record and report the relevant data, and ensure the credibility of all the data related to the clinical trial as well as the reasonableness of its processing, quality control will be implemented at all stages of the data processing, and all the data related to the clinical trial will be handed over to the specialized biostatisticians for data processing and statistical analysis.

5.2 Sample size and the rationale for its determination

Sample size estimation was performed using Power Analysis and Sample Size 15.0 software (NCSS, Kaysville, UT) using a single stage phase II study design. The objective of this study is to evaluate the proportion of patients who achieve CMR with dasatinib in combination with CAR-T cell therapies in adult Ph- positive ALL. The study aimed to improve the CMR rate from 35% after the induction phase to 65% after CD19 CAR-T cell therapy. A total of 27 patients were needed to demonstrate this hypothesis with a power of 90%, a type I error probability of 5% and a drop-out of 10%.

5.3 Significance level and certainty of clinical studies

The significance level α was taken as 0.05 and the degree of certainty 1-β was taken as 0.9.

5.4 Statistical analysis of the population and statistical methods

5.4.1 Statistical analysis of populations

All enrolled patients were included in the safety analysis; all patients who had received CD19 CAR-T cells infusion were included in the effectiveness analysis.

5.4.2 Methods of statistical analysis

All statistical analyses were processed using statistical software to complete the designed set of statistical analyses according to the statistical analysis plan.

The description of quantitative indicators will calculate the mean, standard deviation, median, minimum, and maximum values. Categorical indicators are described by the number of examples and percentages for each category.

OS was defined as the time from treatment initiation to death from any cause. For patients who achieved CHR, LFS was defined as the time from CHR to hematological relapse or death, and CIR was defined as the time from CHR to relapse. Kaplan–Meier curves were plotted for OS and LFS, comparisons between groups were made using the log-rank method. The competing risk method was used to estimate the CIR, with death in the CHR as the competing event. All P-values presented were two-tailed, with values less than 0.05 considered statistically significant.

5.5 Assessment and definition

CHR was defined as <5% bone marrow blasts, no peripheral blood blasts, and no extramedullary disease, together with a neutrophil count >1×10^9/L and a platelet count >100×10^9/L. Hematological relapse was defined as >5% bone marrow blasts, the presence of blasts in the peripheral blood, or the presence of extramedullary disease after a previous CHR. CMR was defined as undetectable BCR/ABL1 transcripts measured by quantitative RT-PCR with a sensitivity of 10^{-4} in the bone marrow. Major molecular remission was defined as a *BCR/ABL1* to *ABL1* ratio of 0.1% or less on the international scale for p210 *BCR/ABL1* or a 3-log reduction in transcripts for p190 *BCR/ABL1*. Molecular relapse was defined as a 2-log or greater increase in the BCR/ABL1 to ABL1 ratio. ABL1 mutations were evaluated at the time of hematological or molecular relapse.

5.6 Handling of missing values and outliers

Handling of missing values: deletion of missing values

Handling of outliers: treat outliers as missing values and handle them according to the missing value handling method

5.7 Expected drop-out rate

10%

6 Data recording and management

6.1 Case report form

A CRF was required for each subject who signed the informed consent form.

The CRF should be completed by the investigator or the investigator's designee with reference to the original case and should not be altered. Any modifications should be made in accordance with the instructions for completing the CRF and signed with the name of the person who made the modification and the date of the modification. The investigator is required to verify the accuracy and authenticity of the information in each CRF and sign the name and

date of verification. The investigator is solely responsible for the accuracy and authenticity of all data entered into the CRF. The Principal Investigator is required to review the CRF for completeness and accuracy and to sign and date the CRF as directed.

The completeness and acceptability of the CRF will be reviewed during the Monitor's periodic visits to the research center. The Collaborator or its delegate shall allow the Supervisor access to study-related medical and hospitalization records to ensure the correctness of the CRF. The completed CRF shall remain entirely the property of the Collaborator and shall not be divulged in any form to third parties without the written consent of the Collaborator, except to authorized representatives of the relevant governmental health or competent authorities.

6.2 Record-keeping

The Investigator agrees to maintain records as agreed upon, which documents include (but are not limited to) study-specific documents, identification record forms for all participating subjects, medical records, temporary media (e.g., thermal paper), original worksheets, all original signed and dated informed consent forms, and detailed medication record forms for evaluation and auditing by the regulatory authorities, the Collaborating Party, or its delegates. The research center should photocopy any source documents printed on biodegradable thermal paper and then file them with the original documents in the subject's medical record to ensure that they remain legible under long-term storage. In addition, Section 4.9.5 of International Conference on Harmonization (ICH) E6 requires investigators to maintain the critical documents specified in ICH E6 (Section 8) until at least 2 years after final approval of the marketing application for the drug indication being studied or, if the application is not approved, until 2 years after the study has been terminated and the regulatory authority has been notified. In addition, study records may be retained for a period of more than 2 years as required by applicable regulations or through agreements with collaborators.

See the Clinical Research Agreement for the Collaborator's record keeping requirements. Investigators should first contact the Collaborator and obtain written approval from the Collaborator before handling any of these documents.

6.3 Source documents

As required by relevant regulations, the investigator should keep the original records of the clinical study appropriately. Investigators must keep trial documents such as study protocols, CRFs, and relevant correspondence with collaborators until 5 years after the end of the study, or until the collaborators notify the investigator that they can destroy these documents.

7 Criteria and procedures for termination of clinical studies

Study discontinuation is the stopping of a clinical study in its entirety halfway when a study that has not been concluded according to the protocol. The main purpose is to protect the rights and interests of the subjects and to ensure the quality of the study.

8 Expected progress of the study

Duration of study enrollment of subjects: 36 months

Study observation period: 2 years

9 Assessment and reporting of adverse events

9.1 Definition of Adverse Events

9.1.1 Adverse Events (AE)

Any adverse medical event not related to the intended therapeutic effect, including uncomfortable symptoms, signs, or abnormal laboratory tests, that occurs during or after treatment of a subject in a clinical study.

Abnormal laboratory values or test results that occur after signing the informed consent form constitute an AE only if they cause clinical signs or symptoms that are clinically significant, require treatment (e.g., hematological abnormalities requiring transfusion or hematopoietic stem cell support), or require a change in the dose of the investigational product.

9.1.2 Serious Adverse Events (SAEs)

A serious adverse event is an adverse event that occurs during any phase of the study (before, during, and during follow-up) that meets one or more of the following criteria: death, fatal illness or injury, re-hospitalization or prolongation of hospitalization, disability or deformity that results in loss of ability to care for oneself, or congenital malformation.

Serious medical event: may jeopardize the subject or may require medical intervention to prevent the above . The investigator will be required to assess each adverse event or complication that meets the above criteria and provide feedback to the study sponsor. The investigator is required to report on the adverse event and its subsequent progress and treatment. All adverse events: any event that does not meet any of the criteria for a serious event will be recognized as non-serious type.

9.1.3 Major adverse events

9.1.3.1 Cytokine release syndrome (CRS)

Cytokine-associated toxicity, also known as cytokine release syndrome, results from intense immune activation. For immunotherapy to produce the desired clinical efficacy, the intensity of immune activation it uses exceeds that which is typically seen in the natural state. Recent studies have indicated that the incidence and intensity of CRS is positively correlated with tumor load, possibly because a larger tumor load can lead to higher levels of intense T-cell activation. CRS symptoms usually appear a few days after the T-cell infusion, but may also appear several weeks later. In summary, the timing of the onset of CRS symptoms coincides with the timing of peak T-cell expansion in the body. CRS can cause a wide range of symptom types with varying degrees of severity. Relevant clinical symptoms include:

1) Complex symptoms: fever (possibly with chills), depression, fatigue, anorexia, muscle pain, arthralgia, nausea, vomiting, headache;

2) Skin: rash;

3) Gastrointestinal: nausea, vomiting, diarrhea;

4) Respiratory: shortness of breath, hypoxemia;

5) Cardiovascular: tachycardia, wide pulse pressure, hypotension, increase in cardiac output (early stage), tendency to decrease cardiac output (late stage);

6) Coagulation: elevated D-dimer, hypofibrinogenemia (may be associated with bleeding);

7) Kidney: azotemia;

8) Liver: transaminitis, hyperbilirubinemia;

9) Nervous system: headache, altered mental status, confusion, delirium, difficulty in conjugating words or aphasia, hallucinations, tremors, impaired distance discrimination, altered gait, epilepsy

10) Complications of CRS with potentially fatal consequences include: cardiac malfunction (cardiac insufficiency, respiratory arrest, malignant arrhythmia), adult respiratory distress syndrome, neurotoxicity, renal and/or hepatic failure, cerebrovascular accidents, disseminated intravascular coagulation, and the occurrence of other unforeseen complications.

CRS grading system

**separate into different kinds**	**Toxicity indicators**
Level 1	No life-threatening critical symptoms, only symptomatic treatment is needed. Examples of symptoms: fever, nausea, fatigue, headache, muscle pain, depression
Level 2	Symptoms requiring moderate treatment: oxygen demand <40% or hypotension relieved by fluids or 1 low dose of vasopressor or grade 2 tissue organ toxicity
Level 3	Symptoms requiring moderate treatment: oxygen demand ⩾40% or hypotension requiring high dose vasopressors or multiple vasopressors or grade 3 tissue organ toxicity or grade 4 transaminase elevation
Level 4	Critical symptoms of life-threatening illness: need for ventilator or grade 4 tissue organ toxicity (excluding elevated transaminases)
Level 5	dead

Note: Adverse events were graded for severity with reference to the Common Terminology Criteria for the Evaluation of Adverse Events (NCI CTCAE 5.0) published by the American Institute for Cancer Research.

Based on clinical outcomes of approved CAR-T products reported in the literature in 2019, the incidence of CRS for CAR-T for B-ALL was 77%, including 46% for grade 3 or higher CRS, and the incidence of CRS for CAR-T for B-NHL was 58%, including 22% for grade 3 or higher CRS.

CRS treatment:

1) For Grade 1 CRS:

a. Prophylactic supportive therapy may be used.

b. Infection assessment: Treat possible fever and neutropenia, monitor fluid balance, and administer antipyretics and analgesics as necessary.

2) For Grade 2 CRS occurring without multiple complications and in non-elderly subjects:

Prophylactic supportive therapy may be used; heart and other organ function may be closely monitored.

3) Grade II with multiple comorbidities or elderly subjects, and Grade III and IV CRS:

a. Prophylactic supportive therapy may be used;

b. Tociluzumab administered over a period of 1 hour or more, and consider repeat administration if clinical symptoms do not resolve within 24-48 hours of administration.

c. Combine with corticosteroid therapy as appropriate. Methylprednisolone 2 mg/kg/day may be used and may be discontinued after a few days of dosing. Dexamethasone (0.5mg/kg, not to exceed 10mg per dose) may be

considered for subjects with signs of severe neurotoxicity. Risk prevention measures include ruling out contraindications, strict adherence to treatment procedures and specifications, close monitoring of vital signs and indicators, and symptomatic treatment at the discretion of physician based on patients' condition.

9.1.3.2 Hypogammaglobulinemia and associated complications

Hypogammaglobulinemia and related complications, such as infections, may occur as a result of the attack of CAR-T cells on the tumor B-cells and the simultaneous depletion of normal B cells. The length of the treatment period for hypogammaglobulinemia is currently inconclusive, and may range from months to years, and may even require lifelong regular treatment.

Response: Immunoglobulin (IVIG) therapy is proposed to restore normal levels serum immunoglobulin levels. It will be at the discretion of the physician with reference to the treatment plan and according to the condition.

9.1.3.3 Infectious complications

Subjects may develop infections with bacteria, mycobacteria, tuberculosis, viruses and some specialized microorganisms in various systems.

Countermeasures: most of the above situations can be relieved on their own or improve with symptomatic treatment, and in rare cases, they may be life-threatening; measures to prevent the risk include ruling out contraindications, strictly following the treatment process and specifications, closely monitoring your vital signs and indicators, and treating symptoms at the doctor's discretion in accordance with the condition.

9.1.3.4 Clinically rare adverse reactions

These include acute kidney injury, cardiac arrest, left ventricular systolic dysfunction, pulmonary edema, respiratory failure, multiorgan failure, speech disorders, prolongation of the QT interval, hypertension, hypoxia, prolongation of the activated partial thromboplastin time, decreased lymphocyte counts, elevated ALTs, elevated blood bilirubin, increased CPKs, hyperglycemia, hyponatremia, ataxia, headache, and tremors.

Countermeasures: most of the above situations can be relieved on their own or improve with symptomatic treatment, and in rare cases, they may be life-threatening; measures to prevent the risk include ruling out contraindications, strictly following the treatment process and specifications, closely monitoring your vital signs and indicators, and treating symptoms at the doctor's discretion in accordance with the condition.

9.1.3.5 Other common clinical adverse reactions

These include neutropenic fever, fever, hypotension, anemia, decreased plate count, leukopenia, increased AST, hypokalemia, and hypophosphatemia;

Countermeasures: most of the above situations can be relieved on their own or improve with symptomatic treatment, and in rare cases, they may be life-threatening; measures to prevent the risk include ruling out contraindications, strictly following the treatment process and specifications, closely monitoring your vital signs and indicators, and treating symptomatic situations at the discretion of your doctor according to your condition.

9.2 Assessment of adverse events

9.2.1 Severity of adverse events

For each adverse event, record the severity or change in severity at each follow-up visit. The following definition of severity can be applied:

**severity**

mildly	Signs or symptoms are detectable by the subject/subject and are easily tolerated
moderately	Symptoms or signs causing discomfort and interfering with daily activities
severe	Subject is incapacitated for work or daily activities

*Note the distinction between Seriousness and Severity of an adverse event. See Serious Adverse Event (SAE) definition for severity of adverse events.

9.3 Recording of adverse events

9.3.1 Recording of general adverse events

New or exacerbated AEs after signing the informed consent form will be documented in the patient's source file. New or exacerbated AEs prior to initiation of CAR-T cell therapy (i.e., lymphodepletion) will need to be documented in the CRF if they meet one of the following criteria:

- All infections
- All clinical AEs ≥ grade 3
- All laboratory abnormalities considered clinically significant by the investigator
- All AEs related to research procedures
- All AEs leading to study termination

Once patients begin lymphodepletion or pre-infusion visits, all new or exacerbated AEs, including laboratory abnormalities deemed clinically significant by the investigator, will be documented in the CRF up to the Month 12 visit.

AE monitoring should continue until the month 24 (EOT) visit. After the month 12 visit and up to the month 24 visit, AE should only be reported to the investigator and recorded in the CRF if one of the following criteria is met:

- Incidents resulting in death
- Incidents are relevant to the research process
- Infection:
- Serious or opportunistic infections. Defined as a bacterial, viral, fungal or parasitic infection that meets one of the following criteria:
    a. Requires anti-infective treatment or
    b. Result in significant disability or hospitalization or
    c. Need for surgery or other interventions
- New onset of neurological disease, or exacerbation of pre-existing neurological disease
- New onset of rheumatic or other autoimmune disease, or exacerbation of a prior rheumatic or other autoimmune disease
- New onset of other hematologic diseases
- Any severe AE or condition that the investigator believes may be associated with CAR-T cell therapy
- Positive replication competent lentivirus (RCL) test result
- Vector insertion site sequencing results have a mono- or oligoclonal pattern or are in the vicinity of

known human oncogenes

- New malignant tumors (T-cell & non-T-cell), except primary malignant tumors
- Progressive multifocal leukoencephalopathy (PML)
- Hepatitis B reactivation

Whenever possible, a diagnosis should be used to describe an AE (including the laboratory abnormalities that comprise the AE) rather than individual underlying signs and symptoms. If a definitive diagnosis cannot be recognized, each sign or symptom should be reported as a separate AE.

AEs will be evaluated according to the Medical Dictionary for Regulatory Agencies (MedDRA) and the Common Terminology Criteria for Adverse Events (CTCAE) version 5.0. If no CTCAE classification exists for a given AE, mild, moderate, severe and life-threatening severity levels will be used, corresponding to grades 1-4. CTCAE grade 5 (resulting in death) will not be used in this study, but is collected as a severity criterion; of course, information about death will be collected through the death form.

The occurrence of AEs should be looked for by non-directional questioning of the patient during the screening process after signing the informed consent form, and at each visit during the study. AEs may also be detected by patient-initiated information during the screening process or between visits, or by physical examination, laboratory tests, or other assessments. Each case of AE should be evaluated, if possible, to determine:

a) Severity Classification (CTCAE v.5.0 Grade 1-4)
b) Duration (start and end dates)
c) Relevance to CAR-T cell therapy (definitely relevant, very probably relevant, probably relevant, probably not relevant, not relevant)
d) Measures taken in response to the study or CAR-T cell therapy (none, suspension of medication, permanent discontinuation, not applicable)
e) Whether medication or treatment is given (no comorbid medication/non-medication, comorbid medication/non-medication)
f) Regression (not recovered, recovered, recovering, recovered with sequelae, lethal, unknown)

All AEs should be treated appropriately. if given combined medication or nonpharmacologic treatment, this measure should be documented in the CRF.

Once an AE is detected, it should be followed until it recovers or is judged to be permanent, and any change in severity, relevance to CAR-T cell therapy, interventions required for treatment, and regression should be assessed at each visit (or more frequently, if necessary).

Progression (including fatal regression) of the primary study indication (i.e., the original disease for which the patient was enrolled for study treatment) should not be reported as an AE, but needs to be documented using appropriate methods.

Data collection for hospitalization events

Patients treated with CAR-T cells require multi-day hospitalization and/or ICU monitoring. These AEs are primarily caused by CRS and MAS, although some may also be caused by lymphodepletion (neutropenic fever, decreased blood counts). CRS/MAS toxicity is an "on-target" effect caused by the expected CAR-T cell expansion, activation, and tumor cell killing.

A typical hospitalization or ICU can generate hundreds of data points and many therapeutic dose changes in a given day. These hospitalization events and days are not scheduled protocol-defined visits, although they are expected to occur in some patients. Hospitalization data collection will be used in this study to systematically collect a subset of patient data to characterize the management of safety events associated with CAR-T cell therapy for the purposes of:

a) Fully inform physicians and patients of the anticipated risks of CAR-T cell therapy and recommended interventions to manage those risks

b) Submission by the Regulator

This was accomplished through targeted collection of combined medication and laboratory data, as well as a CRF specifically designed to capture toxicity, severity, intervention, and post-intervention response/recovery associated with CAR-T cell therapy.

9.3.2 Recording and reporting of serious adverse events

Any SAE that occurs during the screening/pre-treatment phase (from the time the patient signs the informed consent until the time the patient begins study-related treatment) should only be reported to the investigator and should be captured in the CRF and safety database if the event meets at least one of the following criteria:

All events leading to death

All lung or heart abnormalities

All infections

All events related to the research process

Any reportable AE during this study period that also meets severity criteria

Any substantial change in the patient's status that prevents the patient from continuing CAR-T cell therapy (e.g., rapid progression of malignancy, significant decline in physical status)

Any other substantial change in the patient's clinical status, as perceived by the investigators, that may have a potential impact on the patient during lymphodepletion and CAR-T cell therapy

To ensure patient safety, any SAE that occurs during the period after the patient begins study-related therapy (i.e., lymphodepletion) and up to the Month 12 visit, regardless of suspected relevance, must be reported to the investigator within 24 hours of being informed of its occurrence.

Any SAEs occurring after the 12th month visit and up to the 24th month (EOT) visit, during which time they occur, need to be reported to the investigator and documented in the CRF only if one of the following criteria is met:

Incidents resulting in death

Incidents are relevant to the research process

Infection:

Serious or opportunistic infections. Defined as a bacterial, viral, fungal or parasitic infection that meets one of the following criteria:

a. Requires anti-infective treatment OR

b. Result in significant disability or hospitalization or

c. Need for surgery or other interventions

New onset of neurological disease, or exacerbation of pre-existing neurological disease

New onset of rheumatic or other autoimmune disease, or exacerbation of a prior rheumatic or other autoimmune disease

New onset of other hematologic diseases

Any severe AE or condition that the investigator believes may be associated with CAR-T cell therapy

Positive RCL test result

Vector insertion site sequencing results have a mono- or oligoclonal pattern or are in the vicinity of known human oncogenes

New malignant tumors (T-cell & non-T-cell), except primary malignant tumors

PML

Hepatitis B reactivation

Any SAE occurring after the Month 24 (EOT) visit that the investigator suspects to be correlated with CAR-T cell therapy need only be reported to the Yake's drug safety department. Recurrence, complication, or progression of the first SAE must be reported as a follow-up to the first event within 24 hours of the investigator being informed of the follow-up information. SAEs that occur at different time intervals or are otherwise considered completely unrelated to previously reported events should be reported separately as new events.

Collect all SAE-related information and record it on the SAE Report Form; all applicable sections of the form must be completed to provide a clinically complete report. The investigator must assess and document the relevance of each SAE to each specific CAR-T cell treatment (if multiple CAR-T cell treatments are available), complete the SAE report form in Chinese, and submit the completed form to the investigator within 24 hours. For instructions on the SAE submission process and signature requirements, please refer to the investigator folder.

Follow-up information is submitted in the same manner as the initial SAE report. Recurrence, complication, or progression of the first event should be reported as a follow-up of that event, regardless of when it occurred. Follow-up information should describe whether the event has recovered or is still continuing, whether and how it was treated, and whether the patient continued or withdrew from the study.

9.4 Management and follow-up of adverse events

9.4.1 Handling of adverse events

When any adverse event occurs in the study, the investigator should first determine its nature and also take the necessary therapeutic measures to deal with it and to maximize the protection of the subjects' rights and interests.

9.4.2 Costs of handling serious adverse events

In addition to coordinating active treatment and covering the cost of treatment for subjects with serious adverse events related to cellular therapy, the Collaborating Unit will provide legal and financial guarantees to the Investigator, except in cases of medical malpractice.

9.4.3 Follow-up of adverse events

The investigator should follow up all adverse events (including serious adverse events), and may follow up periodically according to the condition until the adverse event has a final outcome, and record the follow-up and the regression of the adverse event.

10 Quality control and quality assurance

10.1 Inspection visits to research centers

Supervision of this trial will be carried out by the investigator or a third party CRO commissioned by the investigator. The supervisor will visit the study center before the start of the study, during the study, and at the end of the study to ensure that all aspects of the protocol have been followed, and will review the source documents to verify the data recorded in the CRF. Source documents are defined as original documents, data and records. Investigators and hospitals should ensure that the Collaborator or its delegate (CRO) and the IRB or IEC have access to the source documents.

All aspects of this study and its documentation were subject to review by the collaborating or commissioning party, including, but not limited to, the investigator's binder, study medications, subjects' medical records, informed consent documents, and review of CRFs and related source documents. It is important that the investigator and other study staff be present at the monitoring visit and that adequate time is available for this work.

The purpose of supervision is to ensure that the rights and interests of subjects in clinical trials are safeguarded, that the data recorded and reported in the trial are accurate, complete and error-free, and that the trial follows the approved protocol and relevant regulations. Supervisors should follow standard operating procedures and supervise the conduct of clinical trials to ensure that they are carried out in accordance with the protocol.

10.2 Protocol Violations

The investigator may not violate the protocol unless it is necessary to eliminate a hazard, i.e., to the research subjects. If other unanticipated circumstances arise that require a change in the procedures set forth in the protocol, the investigator should consult with the collaborating or commissioning party (and the IRB or IEC, if required) to determine the appropriate course of action. For exemptions that are not permitted by the inclusion or exclusion criteria (pre-permitted violations).

The Research Center shall document all protocol violations in the subject's source document and shall notify the Collaborator or its delegate (and the IRB or IEC, if required) if a major violation occurs. Major protocol violations include, but are not limited to, situations involving falsification or misconduct, increased risk to the health of subjects, or confounding of the interpretation of the primary study evaluation. For any major protocol violation, the Research Center shall complete a Protocol Violation Form and have it signed by the Collaborating or Commissioning Party.

10.3 Quality assurance audits

The research center may also be subject to quality assurance audits by the partner or commissioner. In such cases, the partner's designated auditor will contact the research center in advance to schedule an audit visit. The auditor may request a visit to the facilities where laboratory samples are collected, cells are stored and resuscitated, and other facilities used during the study to evaluate whether the test is being performed in accordance with the test protocol, standard operating procedures, and regulatory requirements, and whether the test data are being recorded in a timely, truthful, accurate, and complete manner.

Appendix Treatment of Cytokine Release Syndrome (CRS)

Cytokine release syndrome, which is caused by high intensity immune activation. For immunotherapy to produce the desired clinical efficacy, the intensity of immune activation used will be greater than what is typically seen in the natural state. Recent studies have indicated that the incidence and intensity of CRS is positively correlated with tumor burden, possibly because a larger tumor burden can lead to higher levels of intense T-cell activation. CRS symptoms usually appear a few days after the T-cell infusion, but may also appear several weeks later. In summary, the timing of the onset of CRS symptoms coincides with the timing of peak T-cell expansion in the body. CRS can cause a wide range of symptom types with varying degrees of severity. Relevant clinical symptoms include:

1) Complex symptoms: fever (possibly with chills), depression, fatigue, anorexia, muscle pain, arthralgia, nausea, vomiting, headache;

2) Skin: rash;

3) Gastrointestinal: nausea, vomiting, diarrhea;

4) Respiratory: shortness of breath, hypoxemia;

5) Cardiovascular: tachycardia, wide pulse pressure, hypotension, increase in cardiac output (early stage), tendency to decrease cardiac output (late stage);

6) Coagulation: elevated D-dimer, hypofibrinogenemia (may be associated with bleeding);

7) Kidney: azotemia;

8) Liver: transaminitis, hyperbilirubinemia;

9) Nervous system: headache, altered mental status, confusion, delirium, difficulty in conjugating words or aphasia, hallucinations, tremors, impaired distance discrimination, altered gait, epilepsy

10) Complications of CRS with potentially fatal consequences include: cardiac malfunction (cardiac insufficiency, respiratory arrest, malignant arrhythmia), adult respiratory distress syndrome, neurotoxicity, renal and/or hepatic failure, cerebrovascular accidents, disseminated intravascular coagulation, and the occurrence of other unforeseen complications.

Table 1: CRS treatment principles:

1. Grading the severity of CRS;

2. Adopt different treatment measures for different levels of CRS;

3. Early use of immunosuppressants in patients who present a high risk of CRS while avoiding the effect of immunosuppression on the anti-tumor activity of CAR T cells.

CRS classification	symptom	Treatment
Grade 1	Fever, systemic symptoms	Close observation and supportive therapy<br>Assessment of infections<br>(Fever and neutropenia require treatment, monitoring of fluid balance, and use of antipyretic and analgesic medications as needed.)
Grade 2	Hypotension: requires rehydration or a low dose of vasopressor<br>Hypoxia: <40% O_2<br>Organ toxicity: Grade 2<br>No multiple comorbidities, nonagenarian	Close observation and supportive therapy<br>symptomatic treatment<br>Monitoring of fluid balance<br>Monitoring of heart and other organ function
	Hypotension: requires rehydration or a low dose of vasopressor<br>Hypoxia: <40% O_2<br>Organ toxicity: Grade 2<br>Widespread complication symptoms/elderly	Close observation and supportive therapy<br>symptomatic treatment<br>Monitoring of fluid balance<br>Monitoring of heart and other organ function<br>Tocilizumab±corticosteroids
Grade 3	Hypotension: requires multiple or high doses of vasopressor<br>Hypoxia: ≥40% O_2	Close observation and supportive therapy<br>symptomatic treatment

	Organ toxicity: Grade 3<br>Transaminases: Grade 4	Monitoring of fluid balance<br>Monitoring of heart and other organ function<br>Tocilizumab±corticosteroids
Grade 4	mechanical ventilation<br>Organ toxicity: grade 4 (except transaminases)	Close observation and supportive therapy<br>symptomatic treatment<br>Monitoring of fluid balance<br>Monitoring of heart and other organ function<br>Tocilizumab±corticosteroids

Ref: Current concepts in the diagnosis and management of cytokine release syndrome. Blood. 124(2):188-195.

This appendix is intended for investigators' reference in clinical trials only and is not intended as a mandatory implementation document.

Table 2: Indications and precautions for the use of Tocilizumab in the treatment of CRS

indications	Grade 3 and higher CRS, including but not limited to:<br>1. Echocardiography suggests a left ventricular ejection fraction <40%;<br>2. Elevated blood creatinine more than 2.5 times the pre-CAR T-cell infusion level;<br>3. Regardless of whether norepinephrine administration is intermittent or not, as long as the average dosage administered over a sustained 48-hour period from the first dose is >2 g/min;<br>4. application of norepinephrine could not maintain systolic blood pressure above 90 mmHg;<br>5. oxygen requirement-inhaled oxygen concentration (FiO_2)>50% and continuous oxygen intake for more than 2 hours;<br>6. Severe respiratory distress requiring mechanical ventilation;<br>7. activated partial thromboplastin time (APTT) >2 times the upper limit of normal;<br>8. severe bleeding;<br>9. Creatine kinase > 5 times the upper limit of normal and lasting > 2 days.
recommended dose	8 mg/kg
maximum dose	≤800 mg
Mode of administration	Intravenous infusion for >1 hour.
onset of action	Fever and hypotension usually resolve within a few hours.
Whether multiple administrations are required	Within 24-48 hours of treatment, if symptoms are not relieved, another infusion may be given; or glucocorticoids may be used
Patients with neurological toxicity	Tocilizumab is not recommended and glucocorticoids are recommended.
Possible side effects	Elevated aminotransferases, decreased platelets, elevated cholesterol and LDL, decreased neutrophils, and infections.

Comparison with glucocorticoids	Early onset of action for the treatment of CRS without serious adverse effects on the proliferation and activity of CAR T cells
Drug class	First-line therapeutic agents for the treatment of grade 3 and 4 CRS

References:

Current concepts in the diagnosis and management of cytokine release syndrome. Blood. 124(2):188-195.

Toxicities of chimeric antigen receptor T cells: recognition and management. Blood. 2016. 127(26): 3321-3330.

This appendix is intended for investigators' reference in clinical trials only and is not intended as a mandatory implementation document.

Table 3: Considerations of glucocorticoid drugs for CRS treatment

recommended dose	Methylprednisolone, recommended initial dosage 2 mg/kg/day, usually discontinued after several days of administration
	Dexamethasone, recommended dose 0.5 mg/kg/dose, maximum dosage 10 mg/dose
Those who have been treated with glucocorticoid drugs for a long time	Consider an emergency dose of hydrocortisone
Patients with neurological toxicity	Tocilizumab is not recommended and glucocorticoids are recommended.
side effects	Inhibition of proliferation and anti-tumor activity of CAR T cells
Compared to Tocilizumab	Late onset of therapy for CRS, inhibits CAR T cell proliferation and activity
Drug class	Second-line therapeutic agents for the treatment of grade 3 and 4 CRS

References:

Current concepts in the diagnosis and management of cytokine release syndrome. Blood. 124(2):188-195.

Toxicities of chimeric antigen receptor T cells: recognition and management. Blood. 2016. 127(26): 3321-3330.

This appendix is intended for investigators' reference in clinical trials only and is not intended as a mandatory implementation document.
