# Supplementary material for: Dasatinib and CAR T-Cell Therapy in Newly Diagnosed Philadelphia Chromosome–Positive Acute Lymphoblastic Leukemia: A Nonrandomized Clinical Trial
Source: JAMA Oncol. 2025 Apr 17;11(6):625–9. doi: 10.1001/jamaoncol.2025.0674 (PMC12006910; doi:10.1001/jamaoncol.2025.0674)
Supplement: Supplement 2. — eFigure 1. Trial design eFigure 2. Overall survival and leukemia-free survival eFigure 3. Overall survival and leukemia-free survival, according to the presence or absence of IKZF1 alterations eFigure 4. In vivo kinetics of CAR T cells eTable. Adverse events [file jamaoncol-e250674-s002.pdf]

Supplemental Online Content

Zhang M, Fu S, Feng J, et al. Dasatinib and CAR T-cell therapy in newly diagnosed Philadelphia chromosome–positive acute lymphoblastic leukemia: a nonrandomized clinical trial. *JAMA Oncol*. Published online April 17, 2025.
doi:10.1001/jamaoncol.2025.0674

eMethods. Additional methodological information
eFigure 1. Trial design
eFigure 2. Overall survival and leukemia-free survival
eFigure 3. Overall survival and leukemia-free survival, according to the presence or absence of *IKZF1* alterations
eFigure 4. In vivo kinetics of CAR T cells
eTable. Adverse events

This supplemental material has been provided by the authors to give readers additional information about their work.

© 2025 Zhang M et al. *JAMA Oncology*.

eMethods

CD19 and CD22 CAR construct and CAR T-cell manufacture

Humanized CD19 CARs and CD22 CARs were lentiviral vectors carrying a second generation CAR with 41-BB co-stimulatory and CD3ζ signaling domains (from Shanghai YaKe Biotechnology Ltd., Shanghai, China). The antigen recognition domains of CD19- or CD22-specific CARs are single-chain variable fragments obtained from a human antibody phage display library. Autologous peripheral blood mononuclear cells (PBMCs) were collected from patients through leukapheresis. PBMCs were stimulated with magnetic beads coated with anti- CD3/CD28 antibodies (Thermo Fisher Scientific) overnight. The next day, transduction via a lentiviral vector was performed at a multiplicity of infection 1:8 ratio. Transduced cells were cultured in X-VIVO 15, a serum- free medium (Lonza) with 300 IU/mL interleukin- 2, for the duration of cell culture (7-11 days). CD19 CAR T-cells were manufactured from fresh cells, and CD22 CAR T-cells from cryopreserved cells.

Treatment process

Patients received pretreatment with glucocorticoids (dexamethasone 10 mg intravenously once daily) for 5–7 days. For patients with white blood cell (WBC) counts above 30,000/μL, cyclophosphamide 300 mg/day was also administered intravenously to reduce the leukemia burden, combined with leukapheresis, if necessary. This was followed by a 2-week induction regimen of vindesine and glucocorticoids (vindesine 4 mg intravenously on days 1 and 8, dexamethasone 10 mg intravenously on days 1–11, and 5 mg intravenously on days 12–14). Dasatinib was added to the induction regimen at 100 mg/d. After induction, patients who achieved complete hematological remission (CHR) underwent a single apheresis for CAR T-cell manufacture. CD19 CAR T-cells were manufactured from fresh cells, and CD22 CAR T-cells from cryopreserved cells. Patients received lymphodepleting chemotherapy with fludarabine 30 mg/m^2 daily for three consecutive days (days -4 to -2) and cyclophosphamide 500 mg/m^2 daily for two consecutive days (days -3 to -2), followed by a single infusion of CD19 CAR T-cells. The timing of sequential CD22 CAR T-cell therapy included normal B-cell reappearance or failure to achieve CMR after CD19 CAR T-cell therapy. Patients received the same lymphodepleting chemotherapy of fludarabine and cyclophosphamide before CD22 CAR T-cell infusion. CAR T-cells were administered at a target dose of 2×10^6 CAR$^+$ T-cells/kg. Dasatinib discontinued during CAR T-cell therapy and restarted after the recovery of neutropenia and thrombocytopenia to grade 1 from CAR T-cell therapy. All patients received single-agent dasatinib maintenance therapy (100 mg/d) after sequential CAR T-cell therapy. Central nervous system (CNS) prophylaxis with intrathecal chemotherapy (methotrexate 10 mg, cytarabine 50 mg, and dexamethasone 5 mg) was administered twice, each time on the first day of lymphodepleting chemotherapy.

Definitions

CHR was defined as <5% bone marrow blasts, no peripheral blood blasts, and no extramedullary disease, together with a neutrophil count $>1\times10^9$/L and a platelet count $>100\times10^9$/L. CMR was defined as undetectable BCR/ABL1 transcripts measured by quantitative RT-PCR with a sensitivity of 10^{-4} in the bone marrow. Major molecular remission was defined as a *BCR/ABL1* to *ABL1* ratio of 0.1% or less on the international scale for p210 *BCR/ABL1* or a 3-log reduction in transcripts for p190 *BCR/ABL1*. Hematological relapse was defined as >5% bone marrow blasts, the presence of blasts in the peripheral blood, or the presence of extramedullary disease after a previous CHR. Molecular relapse was defined as a 2-log or greater increase in the *BCR/ABL1* to *ABL1* ratio. *ABL1* mutations were evaluated at the time of hematological or molecular relapse.

In this study, racial categories were defined by the researchers using standard classifications. Participants were classified by the research team to ensure consistency and enable analysis of racial disparities in outcomes. Overall survival (OS) was defined as the time from treatment initiation to death from any cause. Patients who were alive at the time of analysis were censored at the last follow-up date. For patients who achieved CHR, Leukemia-free survival (LFS) was defined as the time from CHR to the first documented hematological relapse or death from any cause, whichever occurred first. Patients who were alive and relapse-free at the time of analysis were censored at the date last know alive with no report of relapse.

© 2025 Zhang M et al. *JAMA Oncology*.

eFigure 1. Trial design

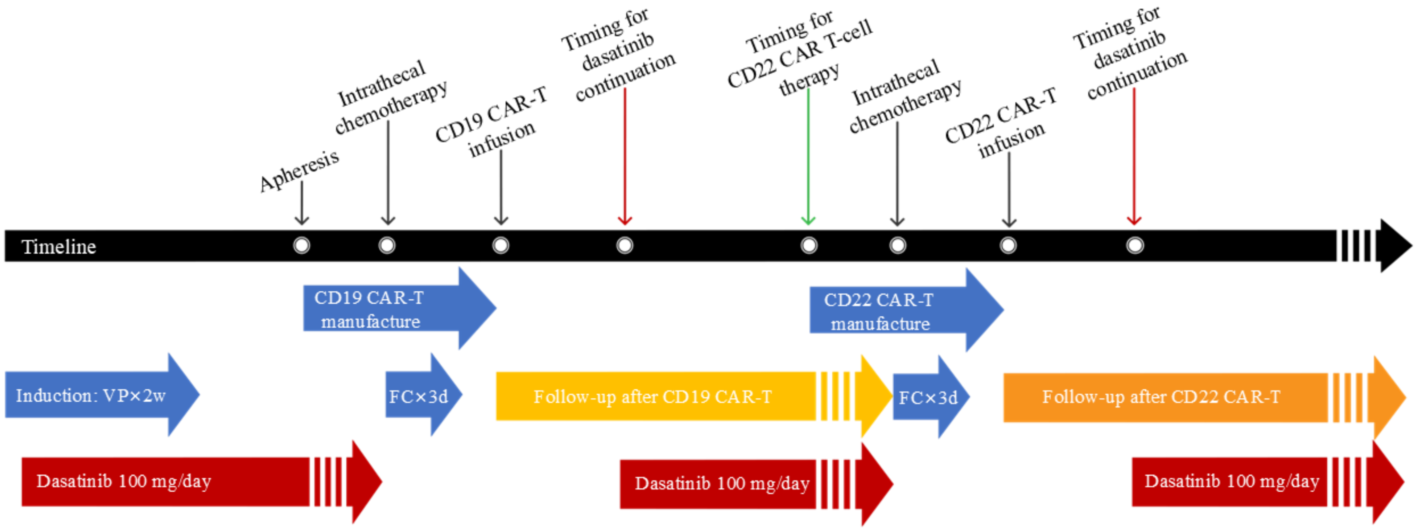

Induction: a 2-week induction regimen of vindesine and glucocorticoids (vindesine 4 mg intravenously on days 1 and 8, dexamethasone 10 mg intravenously on days 1–11, and 5 mg intravenously on days 12–14).
Lymphodepleting chemotherapy: fludarabine 30 mg/m^2 daily for three consecutive days (days -4 to -2) and cyclophosphamide 500 mg/m^2 daily for two consecutive days (days -3 to -2), followed by a single infusion of CAR T-cells (day 0).
Timing for dasatinib continuation: after recovery of neutropenia and thrombocytopenia to grade 1 from CAR T-cell therapy.
Timing for CD22 CAR T-cell therapy: failure to achieve CMR or normal B-cell reappearance after CD19 CAR T-cell therapy.
Intrathecal chemotherapy: administered twice, each time on the first day of lymphodepleting chemotherapy with methotrexate 10 mg, cytarabine 50 mg, and dexamethasone 5 mg.

© 2025 Zhang M et al. *JAMA Oncology*.

eFigure 2. Overall survival and leukemia-free survival.

Shown are the estimated distributions of overall survival (Panel A), and leukemia-free survival (Panel B).

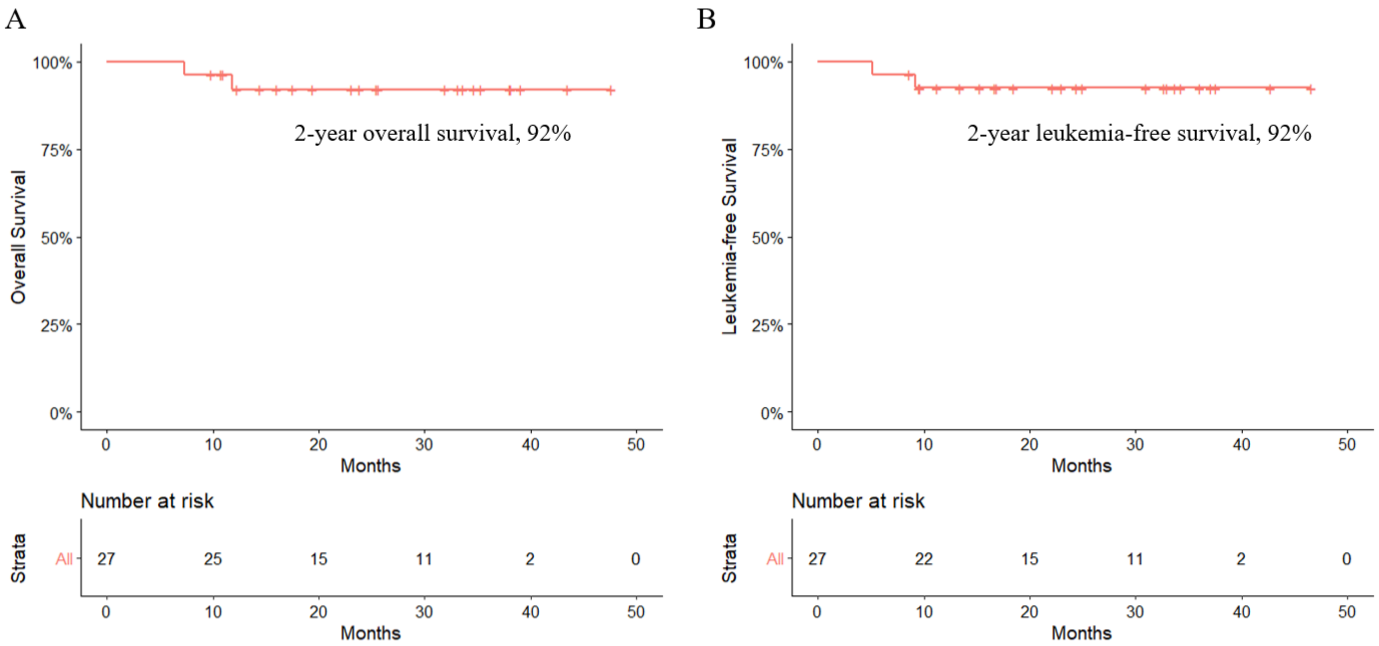

© 2025 Zhang M et al. *JAMA Oncology*.

eFigure 3. Overall survival and leukemia-free survival, according to the presence or absence of *IKZF1* alterations.

Shown are the estimated distributions of overall survival (Panel A), and leukemia-free survival (Panel B), according to the presence or absence of *IKZF1* alterations.

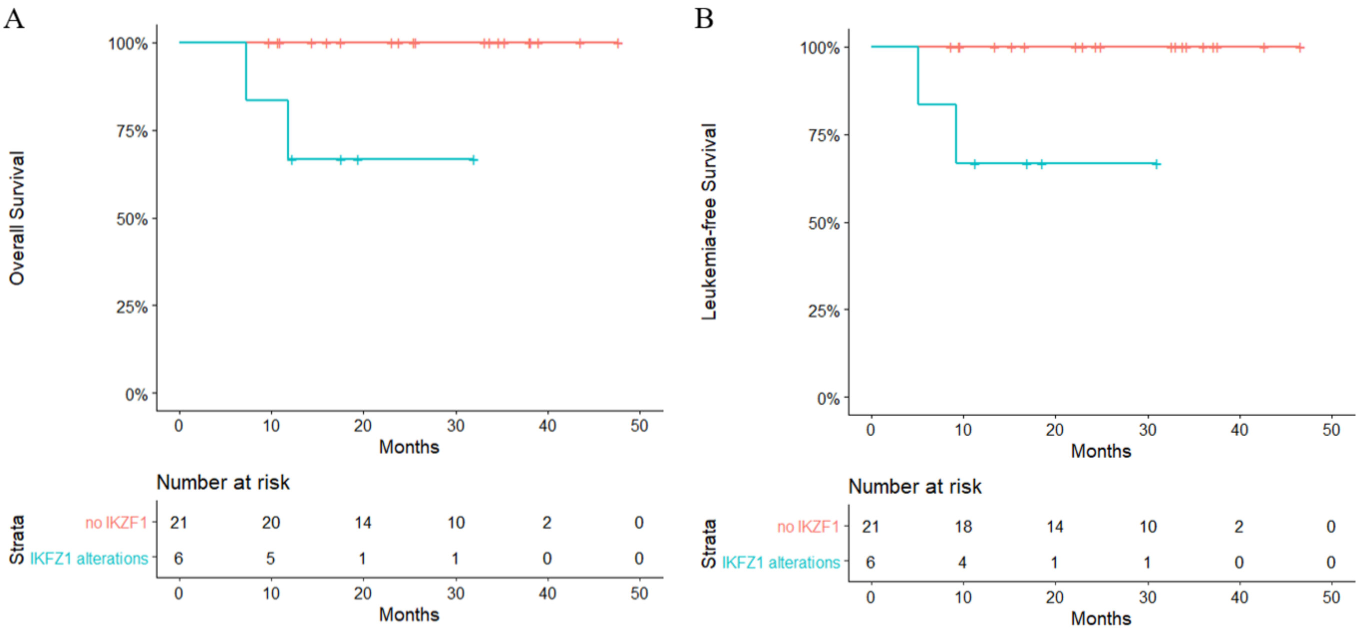

© 2025 Zhang M et al. *JAMA Oncology*.

eFigure 4. In vivo kinetics of CAR T cells.
Shown are in vivo kinetics of CD19 (Panel A) and CD22 (Panel B) CAR T cells.

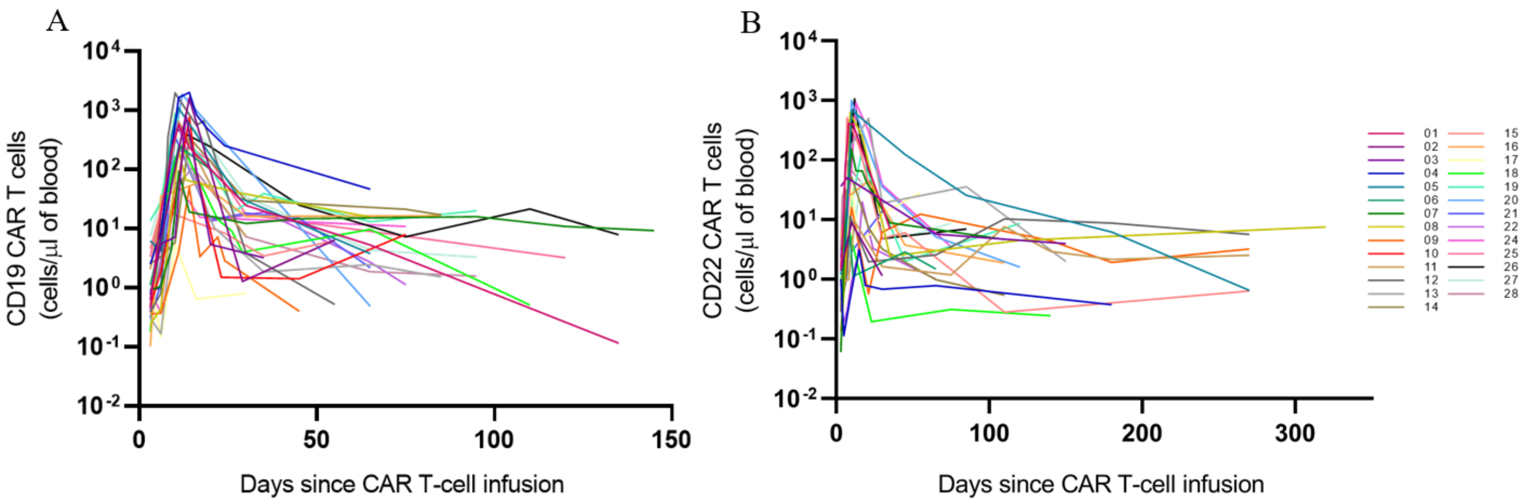

© 2025 Zhang M et al. *JAMA Oncology*.

eTable. Adverse events

	Induction (n=28)				CD19 CAR-T (n=27)				CD22 CAR-T (n=25)			
	Grade 1	Grade 2	Grade 3	Grade 4	Grade 1	Grade 2	Grade 3	Grade 4	Grade 1	Grade 2	Grade 3	Grade 4
Leukopenia	1	0	9	12	1	12	9	4	2	8	4	10
Neutropenia	1	1	5	14	0	5	11	7	3	3	4	11
Lymphopenia	2	1	9	7	0	0	5	22	0	0	3	22
Anemia	1	1	12	0	1	4	5	0	3	7	0	0
Thrombocytopenia	1	0	3	5	1	0	1	0	3	3	0	0
Increased ALT	8	1	2	0	3	1	1	0	5	0	1	0
Increased AST	4	1	0	0	2	0	1	0	2	0	0	0
Rash	1	1	1	0	0	1	0	0	0	0	0	0
Pulmonary infection	0	0	1	0	0	0	0	0	0	0	0	0
Urinary tract infection	0	0	1	0	0	0	0	0	0	1	0	0
Bacterial infection	0	1	0	0	0	0	0	0	0	0	0	0
Nausea	0	0	0	0	6	1	0	0	4	2	0	0
Vomiting	0	0	0	0	2	4	0	0	2	2	0	0
Diarrhea	0	0	0	0	1	0	0	0	0	1	0	0
Productive cough	1	1	0	0	1	0	0	0	0	0	0	0
Atrial fibrillation	0	0	0	0	0	0	0	0	0	1	0	0
Systemic oedema	1	1	0	0	0	0	0	0	0	0	0	0
Pleural effusion	1	1	0	0	0	0	0	0	0	0	0	0
CRS	NA	NA	NA	NA	9	0	0	0	12	0	0	0
ICANS	NA	NA	NA	NA	0	0	0	0	0	0	0	0

ALT= alanine aminotransferase; AST= aspartate aminotransferase; CRS=cytokine release syndrome; ICANS=Immune effector cell–associated neurotoxicity syndrome; CAR=chimeric antigen receptor

© 2025 Zhang M et al. *JAMA Oncology*.
