# Supplementary material for: Dasatinib and CAR T-Cell Therapy in Newly Diagnosed Philadelphia Chromosome–Positive Acute Lymphoblastic Leukemia: A Nonrandomized Clinical Trial
Source: JAMA Oncol. 2025 Apr 17;11(6):625–9. doi: 10.1001/jamaoncol.2025.0674 (PMC12006910; doi:10.1001/jamaoncol.2025.0674)
Supplement: Supplement 3. — Data sharing statement [file jamaoncol-e250674-s003.pdf]

Data Sharing Statement

Zhang. Dasatinib and CAR T-Cell Therapy in Newly Diagnosed Philadelphia Chromosome–Positive Acute Lymphoblastic Leukemia. *JAMA Oncol*. Published April 17, 2025. doi:10.1001/jamaoncol.2025.0674

Data
Additional Information: This trial was registered at www.clinicaltrials.gov as #NCT04788472.
Data available: Yes
Data types: Deidentified participant data
How to access data: Any request for data sharing should be directed to the corresponding author via huanghe@zju.edu.cn.
When available: With publication

Supporting Documents
Document types: None

Additional Information
Who can access the data: researchers whose proposed use of the data has been approved
Types of analyses: for any purpose
Mechanisms of data availability: after approval of a proposal
